# Linking Megalin, Cubilin, Caveolin-1, GIPC1 and Dab2IP Expression to Ocular Tumorigenesis: Profiles in Retinoblastoma, Choroidal Melanoma, and the Normal Human Eye

**DOI:** 10.3390/cancers17233785

**Published:** 2025-11-26

**Authors:** Petra Kovačević, Petar Todorović, Nela Kelam, Suzana Konjevoda, Nenad Kunac, Josipa Marin Lovrić, Katarina Vukojević

**Affiliations:** 1 Department of Ophthalmology, University Hospital Center of Zagreb, 10000 Zagreb, Croatia; petra.kovacevic1@kbc-zagreb.hr; 2 Department of Anatomy, Histology and Embryology, University of Split School of Medicine, Šoltanska 2A, 21000 Split, Croatia; petar.todorovic@mefst.hr (P.T.); nela.kelam@mefst.hr (N.K.); 3 Center for Translational Research in Biomedicine, University of Split School of Medicine, Šoltanska 2A, 21000 Split, Croatia; 4 Department of Ophthalmology, General Hospital Zadar, 23000 Zadar, Croatia; suzana.konjevoda@gmail.com; 5 Department of Health Studies, University of Zadar, 23000 Zadar, Croatia; 6 Department of Pathology, Forensic Medicine and Cytology, University Hospital Centre Split, Spinčićeva 1, 21000 Split, Croatia; nenad.kunac@mefst.hr; 7 Department of Ophthalmology, University Hospital Centre Split, Spinčićeva 1, 21000 Split, Croatia; josipa.marin@mefst.hr; 8 Department of Anatomy, School of Medicine, University of Mostar, 88000 Mostar, Bosnia and Herzegovina; 9 Mediterranean Institute for Life Sciences (MedILS), University of Split, Meštrovićevo Šetalište 45, 21000 Split, Croatia

**Keywords:** uveal melanoma, retinoblastoma, receptor-mediated endocytosis, megalin, cubilin, Caveolin-1, GIPC1, DAB2IP, immunofluorescence

## Abstract

**Background/Objectives**: Retinoblastoma (RB) and uveal melanoma (UM) remain vision-threatening and lethal ocular malignancies with limited molecular markers of differentiation state and prognosis. We investigated whether proteins governing endocytosis and signaling, including Megalin (LRP2), Cubilin (CUBN), Caveolin-1, GAIP-interacting protein C-terminus 1 (GIPC1), and Disabled homolog 2-interacting protein (DAB2IP), exhibit subtype-specific expression patterns in ocular tumors and whether these patterns are related to transcriptomic profiles and survival. **Methods**: Formalin-fixed, paraffin-embedded human ocular tissues included controls (*n* = 10), retinoblastoma (*n* = 10), and UM subtypes (epithelioid, spindle, mixoid; total *n* = 30). Immunofluorescence for LRP2, CUBN, CAV1, GIPC1, and DAB2IP was quantified using ImageJ (version 1.54g) across standardized high-power fields; per-specimen means were used for statistical analysis (Shapiro–Wilk test; one-way ANOVA with Tukey’s post hoc test). Public data analyses comprised: (i) overall survival in TCGA-UVM using GEPIA2; (ii) differential expression in GEO datasets (GSE62075: melanocytes vs. UM cell lines; GSE208143: retinoblastoma vs. pediatric control retina) and (iii) multivariate Cox proportional hazards regression analysis using the GEPIA3 online platform. **Results**: LRP2 expression was uniformly reduced across retinoblastoma and all UM subtypes versus control. CUBN expression decreased in retinoblastoma and epithelioid melanoma, was retained in spindle melanoma, and increased in mixoid-cell melanoma. CAV1 expression was increased in epithelioid melanoma but reduced in retinoblastoma, mixoid, and spindle melanomas. GIPC1 and DAB2IP expression were preserved in epithelioid melanoma yet significantly reduced in retinoblastoma and mixoid/spindle melanomas. In TCGA-UVM, higher *CAV1* and *GIPC1* mRNA expression was associated with worse overall survival (*p* ≈ 0.025 and 0.036), whereas *LRP2*, *CUBN*, and *DAB2IP* expression were not significant. GEO analyses revealed no significant differences for the five genes in UM cell lines versus melanocytes (GSE62075). However, in retinoblastoma (GSE208143), *LRP2* was downregulated, while *CUBN*, *CAV1*, *GIPC1*, and *DAB2IP* were upregulated. **Conclusions**: Endocytic/signaling proteins exhibit distinct, subtype-linked expression in ocular tumors. Integration with public datasets highlights *CAV1* and *GIPC1* as adverse survival correlates in UM and positions LRP2/CUBN/DAB2IP dysregulation as features of ocular tumor biology, nominating candidate biomarkers and mechanistic targets.

## Simple Summary

Eye cancers can damage vision and, in some cases, threaten life. Retinoblastoma mainly affects children, while uveal melanoma is the most common eye cancer in adults. Doctors need better ways to judge how aggressive these tumors are and to find new treatment targets. We examined five proteins that help cells handle nutrients and signals, Megalin, Cubilin, Caveolin-1, GIPC1, and DAB2IP, in normal eye tissue, retinoblastoma, and different forms of choroidal melanoma. Using fluorescent staining, we measured the amount of each protein present, then compared our results with publicly available gene data and patient survival information. Our goal was to learn whether changes in these proteins track with tumor type and outcome. These findings may support the development of future tools for diagnosis, risk prediction, and therapy design in eye cancer.

## 1. Introduction

Ocular malignancies are among the most challenging tumors to diagnose and treat due to their location, potential to compromise vision, and tendency to metastasize [1,2]. Retinoblastoma (RB), the most common intraocular cancer in children, arises from retinal progenitor cells that carry biallelic *RB1* inactivation and is characterized by rapid proliferation, poorly differentiated histology, and a propensity for extraocular spread if left untreated [3,4]. In contrast, uveal melanoma, the most common primary intraocular malignancy in adults, develops from melanocytes within the choroid, ciliary body, or iris, and remains the leading cause of cancer-related death due to ocular tumors [1].

Histopathological subtypes of choroidal melanoma (CM), including epithelioid, spindle, and myxoid, differ in their morphology, biological behavior, and prognosis, with epithelioid melanomas generally associated with poorer survival outcomes [5,6]. Despite progress in genetics and imaging, the molecular determinants underlying ocular tumorigenesis and subtype-specific behavior remain incompletely understood [7].

Dysregulated endocytosis and nutrient trafficking contribute to tumor initiation and progression [8]. Megalin (LRP2) and Cubilin (CUBN) are multiligand endocytic receptors essential for epithelial transport of carrier-bound nutrients and proteins, including intrinsic factor–vitamin B12, albumin, and vitamin D–binding protein [9,10]. As co-receptors, LRP2–CUBN mediate albumin/lipoprotein uptake in absorptive epithelia [11,12]. Altered expression links to dedifferentiation and outcomes in human carcinomas [13]. Yet their distribution and function in ocular tumors remain insufficiently defined, despite LRP2-dependent signaling control and critical endocytic homeostasis in the eye [14,15].

LRP2 is an LDL-receptor–family endocytic receptor, identified as gp330 in rat kidney [16]. It localizes apically in absorptive epithelia to internalize ligands (albumin, lipoproteins) and cooperates with CUBN for nutrient uptake [11,12]. In cancer, LRP2 exhibits context dependence: melanomas acquire LRP2, and its knockdown curtails proliferation, whereas several epithelial cancers epigenetically silence it, linking low expression to dedifferentiation and poor survival [8]. In ocular tissues (RPE, ciliary body), LRP2 is expressed and modulates Sonic Hedgehog activity at the retinal margin, implicating ocular tumorigenesis [14,15].

Cubilin is a large peripheral endocytic receptor that binds intrinsic factor–vitamin B12 and associates with the transmembrane receptor LRP2 [9,10]. Megalin-Cubilin cooperativity mediates epithelial uptake of carrier-bound proteins, including albumin and vitamin D-binding protein [10]. Altered *CUBN* expression carries prognostic significance in carcinomas; loss of CUBN predicts worse outcomes in clear-cell renal cell carcinoma [13]. In contrast, membrane CUBN positivity predicts a better response and survival with tyrosine kinase inhibitors [13,17]. CUBN overexpression has also been linked to adverse prognosis in colorectal cancer [18]. These roles motivate the evaluation of CUBN as a context-dependent determinant of nutrient handling and tumor behavior in ocular malignancies.

Caveolin-1 (CAV1) is the principal structural protein of caveolae, plasma-membrane invaginations involved in endocytosis, mechanotransduction, lipid homeostasis, and signal transduction [19,20,21]. In cancer, CAV1 exhibits striking context dependence: in some settings, it suppresses primary tumor growth, whereas in others, it enhances invasion and metastasis, depending on the cell type, stage, and microenvironment [22,23]. In melanoma specifically, CAV1 has been implicated in promoting metastasis in experimental models and is associated with metastatic risk in patients [22]. Given this duality, profiling CAV1 in ocular tumors is warranted, as primary studies in uveal melanoma have linked CAV1 expression to PI3K signaling and vasculogenic mimicry, and have shown distinct expression patterns compared to those in cutaneous melanoma [24].

Beyond endocytic receptors and membrane scaffolds, GIPC1 (GAIP-interacting protein, C terminus 1) serves as an adaptor, linking transmembrane receptors to trafficking and cytoskeletal machinery [25,26]. It modulates receptor recycling and signaling via the PDGFR/PI3K–AKT and neuropilin–RhoA pathways, shaping proliferation, motility, and invasion [27]. GIPC1 is overexpressed and functional in breast, ovarian, and pancreatic cancer models, promoting growth and migration [28,29]. In ocular tumors, evidence remains sparse: uveal melanoma studies implicate the NRP1–GIPC1 axis without systematic expression analyses, and most ocular studies focus on developmental roles rather than malignancy [30].

Lastly, DAB2IP (Disabled homolog 2–interacting protein) is a tumor suppressor that restrains PI3K–AKT, NF-κB, and ERK signaling, regulating apoptosis, epithelial–mesenchymal transition (EMT), and invasion across various cancers, including prostate, colorectal, and gastric [31,32,33]. Epigenetic silencing by promoter methylation or repression by oncogenic microRNAs commonly reduces DAB2IP expression, correlating with dedifferentiation, therapy resistance, and poorer outcomes [34,35]. Despite this broad evidence, the expression patterns, functional impact, and prognostic value of this gene in ocular tumors remain poorly characterized, warranting a systematic evaluation in RB and uveal melanoma [36].

Collectively, Megalin–Cubilin endocytosis, CAV1-mediated membrane signaling, and adaptor/tumor-suppressor nodes (GIPC1, DAB2IP) define axes that may govern differentiation, metabolic supply, and invasion in ocular tumors. Yet their ocular roles remain incompletely mapped. Here, we profile LRP2, CUBN, CAV1, GIPC1, and DAB2IP across normal human ocular tissues, RB, and histopathological subtypes of uveal (choroidal) melanoma, integrating protein expression with publicly available transcriptomic and survival datasets. We investigate whether subtype-specific gains or losses correlate with histopathology and clinical outcomes.

## 2. Materials and Methods

### 2.1. Tissue Samples and Preparation

Formalin-fixed, paraffin-embedded (FFPE) human eye tissues were obtained from the Department of Pathology, Forensic Medicine and Cytology, University Hospital Centre Split. Samples included histologically normal controls (*n* = 10) and pathological specimens diagnosed with RB (*n* = 10) or melanoma subtypes (epithelial, mixoid, spindle; *n* = 30). Control tissues were derived from enucleated eyes for non-neoplastic conditions or from histologically normal regions of eyes removed for RB or melanoma, confirmed by a pathologist. The study was performed with the approval of the Ethics Committee of the University Hospital of Split (protocol code: class 500-03/22-01/47; No: 2181-147/01/06/M.S.-22-02 and date of approval 31 March 2022) in accordance with the Declaration of Helsinki and its updates [37].

Following surgical excision, tissue specimens were immediately immersed in 4% paraformaldehyde dissolved in phosphate-buffered saline (PBS) and fixed for a minimum of 24 h at room temperature. Standard histological processing was performed, including sequential dehydration in increasing concentrations of ethanol, clearance in xylene, and embedding in paraffin wax. Paraffin-embedded blocks were sectioned at 5 µm in a transverse plane using a rotary microtome (RM2125 RTS, Leica, Buffalo Grove, IL, USA) and mounted onto positively charged glass slides to ensure optimal adhesion. Prior to immunohistochemical analysis, slides were stored at room temperature in a controlled dry environment to preserve tissue morphology and antigenicity. Appropriate tissue preservation was confirmed through hematoxylin-eosin (H&E) staining of every tenth segment.

RB specimens were analyzed for *RB1* mutations, whereas melanoma samples were screened for *BRAFV600E*, *BRCA2*, *CDK4*, *CDKN2A*, *PTEN*, and *TP53* mutations. Genetic screening was performed using Sanger sequencing for *BRAFV600E* and *TP53* hotspot regions, and PCR-based assays with allele-specific primers for the remaining genes. Sequence chromatograms were evaluated using Chromas software (version 2.6.6; Technelysium Pty Ltd., South Brisbane, Australia) and mutations with a variant allele frequency ≥ 20% were considered positive. No FISH or NGS methods were used in this study. None of the analyzed samples carried any of the tested mutations. Only well-preserved tissue, verified by two independent pathologists through external and microscopic examination, was selected for analysis, while macerated or poorly preserved material was excluded (3 of 50 total samples, 6% exclusion rate). Inter-pathologist concordance regarding tissue quality was 100%. To further confirm tissue integrity, each block underwent standard hematoxylin and eosin staining prior to inclusion in the study.

### 2.2. Immunofluorescence Staining

Immunofluorescence staining was performed according to established protocols [38,39,40]. Briefly, tissue sections were deparaffinized in xylene (three washes, 10 min each) and rehydrated through descending ethanol concentrations (100%, 96%, 70%) before being immersed in distilled water [41]. Heat-induced epitope retrieval was performed using 0.01 M citrate buffer (pH 6.0) at 95 °C for 30 min in a water steamer. Sections were first treated with a protein blocking solution (ab64226, Abcam, Cambridge, UK) for 20 min, after which they were washed in 0.1 M PBS to minimize nonspecific binding. The primary antibodies (Table 1) were then applied and incubated overnight in a humidity chamber (StainTray slide staining system; Sigma-Aldrich, St. Louis, MO, USA). The following day, after PBS rinsing, sections were incubated for 1.5 h with the appropriate secondary antibodies (Table 1). Nuclear staining was performed with DAPI (4′,6-diamidino-2-phenylindole) following an additional PBS wash. Finally, the samples were air-dried and mounted with coverslips using Immuno-Mount (Thermo Shandon, Pittsburgh, PA, USA).

To determine the specificity of the immunofluorescent staining and reduce nonspecific background, isotype-matched controls, secondary-only controls, and positive controls were employed. In the isotype-matched controls, the primary antibody was replaced with a non-target antibody of the same isotype to assess possible nonspecific binding. Secondary-only controls, which did not include the primary antibody, were utilized to find any interactions that were not specific to the secondary antibody (Supplementary Figure S1). Positive controls were included for each antibody using tissues with established expression patterns: postnatal day 4 (P4) wild-type mouse kidney cortex for LRP2, CUBN (Supplementary Figure S2) and CAV1, GIPC1, and DAB2IP (Supplementary Figure S3). All antibodies were validated on formalin-fixed, paraffin-embedded (FFPE) human tissue following optimized heat-induced epitope retrieval to ensure compatibility and cross-reactivity.

### 2.3. Data Acquisition

Histological slides were examined using a bright-field light microscope (CX43, Olympus, Tokyo, Japan). Immunohistochemically stained sections were captured using an Olympus BX51 microscope equipped with a Nikon DS-Ri2 camera (Nikon Corporation, Tokyo, Japan) and NIS-Elements F software (version 5.22.00). All microphotographs were taken at ×40 magnification. Signal intensity calibration was standardized across all imaging sessions by using fixed exposure times, gain settings, and white-balance parameters [38].

For each tissue specimen, the expression of Megalin, Cubilin, Caveolin-1, GIPC1, and DAB2IP was assessed in ten representative high-power fields (HPFs). HPFs were selected by three blinded investigators who were unaware of the sample diagnosis to minimize sampling bias. Field selection was guided by a digital grid-overlay algorithm within NIS-Elements AR software (version 4.60; Nikon Instruments, Tokyo, Japan), ensuring random and evenly distributed sampling across tumor and adjacent non-tumorous regions.

### 2.4. Image Analysis of Immunohistochemical Staining

Image processing was performed using ImageJ software version 1.54g (National Institutes of Health, Bethesda, MD, USA) and Adobe Photoshop version 21.0.2 (Adobe, San Jose, CA, USA), following previously established and validated protocols (Supplementary Figure S4) [42]. Each captured microphotograph was processed as follows: The red counter-signal was subtracted from the green fluorescence to reduce fluorescence spillage. A median filter with a 7.0-pixel radius was applied to duplicated images. By subtracting the filtered images from the originals, the positive signal was isolated. The processed images were subsequently converted to an 8-bit format and subjected to threshold adjustment using the triangle thresholding algorithm. The “Analyse Particles” function was employed to quantify fluorescence and ascertain the percentage area of fluorescence. In summary, the “area percentage” represents the percentage of the image occupied by the positive fluorescent signal, calculated by dividing the total number of pixels in the image by the number of fluorescent pixels that exceed a specified threshold. The average of the results was calculated for each group that was examined. In regions where tissue folding, damage, or tissue absence occurred, the calculated staining percentage was adjusted accordingly. This correction involved measuring the total number of pixels per image and subtracting the area corresponding to empty space using the Magic Wand tool in Adobe Photoshop [38,43]. The corrected percentage was used for all statistical analyses.

To minimize bias, the same three blinded histologists from the Department of Anatomy, Histology and Embryology (each with more than 20 years of experience) independently analyzed the stained sections and set thresholds based on negative-control images, without knowledge of case identity or experimental group [44]. Interobserver reliability was confirmed using intraclass correlation analysis, with a coefficient exceeding 0.8, indicating a high level of agreement [44].

### 2.5. Statistical Analysis

Statistical analyses were conducted using GraphPad Prism version 9.0.0 (GraphPad Software, San Diego, CA, USA). Data are presented as mean ± standard deviation (SD). The Shapiro–Wilk test was used to assess the normality of the data [45]. For comparative analysis, the percentage of positively stained cells for Megalin, Cubilin, Caveolin-1, GIPC1, and DAB2IP was analyzed across different groups (control, RB, epithelial melanoma, mixoid melanoma, and spindle melanoma) using one-way analysis of variance (ANOVA) followed by Tukey’s post hoc test to assess pairwise differences [45]. A *p*-value < 0.05 was considered statistically significant.

Prior to data collection, a power analysis was performed using G*Power 3.1 software to determine adequate group sizes. Assuming an effect size (f) of 0.6, α = 0.05, and power (1 − β) = 0.8, the required minimum sample per group was estimated at *n* = 8. Our final group sizes (*n* = 10 for control and RB, *n* = 30 for melanoma subtypes combined) therefore provided sufficient statistical power to detect biologically meaningful differences in protein expression.

Clinical covariates (age, sex, and anatomical site) were examined for availability; however, these data were incomplete across archival cases and thus were not included in the statistical model. To minimize potential confounding, all samples were processed and analyzed under identical experimental conditions, and group comparisons were based solely on histopathological subtype.

All graphs were generated using GraphPad Prism 9.0.0, and composite image plates were constructed using Adobe Photoshop 21.0.2. Microphotographs were optimized for visualization by applying background correction and contrast enhancement while preserving the original staining characteristics.

### 2.6. Transcriptomics

Survival analysis based on the expression status of *Megalin* (*LRP2*), *Cubilin* (*CUBN*), *Caveolin 1* (*CAV1*), *GIPC PDZ domain containing family*, *member 1* (*GIPC1*) and *Disabled homolog 2-interacting protein* (*DAB2IP*), was performed using the standard processing pipeline of the publicly available database Gene Expression Profiling Interactive Analysis 2 (GEPIA2, http://gepia2.cancer-pku.cn/, accessed on 30 July 2025) The data sources for the analyses performed in GEPIA2 were the GDC TCGA Ocular Melanomas (UVMs) dataset. Differential expression analysis was performed using one-way ANOVA with cutoff values |log2FC| ≥ 1 and *p* < 0.01. Overall survival analysis was performed based on the expression of the analyzed genes, comparing the lowest and highest 50% for each gene using the Log-rank test with a significance level set at *p* < 0.05. The Kaplan–Meier curves of overall survival were constructed in GEPIA2.

Patients were stratified into two groups (high and low expression) based on the median expression level for each gene. Overall survival (OS) was compared between groups using the Log-rank test, with *p* < 0.05 considered statistically significant. Differential expression analysis in GEPIA2 was conducted using one-way ANOVA, with cutoffs of |log2 fold change| ≥ 1 and *p* < 0.01. Box plots depicting gene expression levels were generated using the GEPIA2 interface.

Hazard ratios (HRs) and confidence intervals for univariate survival analysis were extracted from GEPIA2 output. When GEPIA2 did not provide explicit 95% confidence intervals, these were calculated manually using standard asymptotic methods: SE(log HR) = |log(HR)|/Z, where Z corresponds to the critical value derived from the reported *p*-value, and 95% CI = exp(log(HR) ± 1.96 × SE(log HR)). Differential expression analysis within GEPIA2 was conducted using one-way ANOVA, applying cutoff thresholds of |log2 fold change| ≥ 1 and *p* < 0.01. Box plots depicting gene expression levels were generated using the GEPIA2 interface.

To minimize potential batch effects and technical variability inherent to public datasets, the GEPIA2 and GEPIA3 platforms apply standardized data normalization and quality-control procedures directly integrated from TCGA and GTEx pipelines. All expression data were pre-processed using log2 (TPM + 1) normalization, and batch-effect correction was performed within the TCGA harmonized data framework using the UCSC Xena processing pipeline. Technical replicates and duplicate samples are automatically identified and removed by the GEPIA algorithm prior to differential expression and survival analyses, ensuring that results are based on independent, quality-controlled data. To minimize potential batch effects and technical variability inherent to public datasets, the GEPIA2 and GEPIA3 platforms apply standardized data normalization and quality-control procedures directly integrated from TCGA and GTEx pipelines. All expression data were pre-processed using log2 (TPM + 1) normalization, and batch-effect correction was performed within the TCGA harmonized data framework using the UCSC Xena processing pipeline. Technical replicates and duplicate samples are automatically identified and removed by the GEPIA algorithm prior to differential expression and survival analyses, ensuring that results are based on independent, quality-controlled data. Multivariate Cox proportional hazards regression analysis was performed using the GEPIA3 online platform (https://gepia3.bioinfoliu.com/) to assess the prognostic impact of gene expression on overall survival (OS) in patients with uveal melanoma from the TCGA-UVM cohort. The analysis focused on the expression levels of *LRP2*, *CUBN*, *DAB2IP*, *GIPC1*, and *CAV1*. Hazard ratios (HRs) with corresponding 95% confidence intervals (CIs) were generated, and the Log-rank test was applied to assess statistical significance. Multivariate modeling enabled assessment of whether each gene served as an independent prognostic factor, after adjusting for clinical variables available in the GEPIA3 framework, including patient age, gender, and tumor stage. A forest plot summarizing HRs and CIs were generated and downloaded from the GEPIA3 interface.

### 2.7. Differential Gene Expression Analysis

The National Center for Biotechnology Information’s Gene Expression Omnibus (GEO) database houses datasets from various experiments, allowing users to download gene expression profiles [46]. In our search for GEO datasets with related gene expression profiles, we used the keywords “uveal melanoma”, “Homo sapiens”, and “Expression profiling by array”, yielding 32 available studies. We selected the GSE62075 series (Characterization of the human a9 integrin subunit gene: promoter analysis and transcriptional regulation), which includes the gene expression data from 32 samples, featuring Primary cultures of human corneal epithelial cells (HCECs; number of replicates: 8), human skin epithelial cells (HSEC; number of replicates: 4), human corneal fibroblast cells (HCFCs; number of replicates: 3), human skin fibroblast cells (HSFCs; number of replicates: 3), human uveal melanocytes (UVM; number of replicates: 3), and various human uveal melanoma cell lines (T115, T142 and T143; number of replicates: 2–3) [47]. We analyzed two groups: three primary human uveal melanocyte cultures and three low-passage uveal melanoma cell lines (T115, T142, T143). We discarded the remaining samples, as this was not the focus of our research. RNA was extracted from the cell lines, and gene expression profiling was performed using the Agilent SurePrint G3 Human GE 8 × 60 K microarray platform. Samples were labeled with Cy3, hybridized overnight, and scanned using the Agilent SureScanner (Agilent Technologies, Santa Clara, CA, USA). Raw data were processed with Agilent Feature Extraction software (version 11.5.1.1; Agilent Technologies, Santa Clara, CA, USA), and differential gene expression was analyzed using the LIMMA package in R. To analyze the raw gene expression data, we utilized the online statistical tool GEO2R [46]. The Benjamini and Hochberg (false discovery rate) method was used to calculate adjusted *p*-values. The limma precision weights (vooma) function and quantile normalization (limma package version 3.28.14) were applied to the expression data. RNA sample–level quality control was verified using the parameters reported in the original GEO submissions. For GSE62075, all samples met the quality criteria established by the depositing authors, including RNA integrity numbers (RIN ≥ 7), A260/280 absorbance ratios between 1.9 and 2.1, and passing Agilent spike-in hybridization controls. For GSE208143, RIN values (7.2–9.1), A260/280 ratios (1.8–2.1), and microarray hybridization performance metrics (background intensity, signal distribution, and spike-in controls) were confirmed from the GEO metadata and Supplementary QC files. All included samples satisfied standard Agilent microarray QC thresholds and were suitable for downstream differential expression analysis.

To identify genes that are significantly differentially expressed in the dataset, we applied the following criteria: |log2 (fold change)| > 1 and *p* < 0.05. Upregulated genes were identified with log2FC ≥ 1, while down-regulated genes were determined by log2FC ≤ −1. This log2FC cutoff (≥1/≤−1), corresponding to a ≥2-fold difference in expression, was defined a priori based on established conventions in microarray and transcriptomic analyses using LIMMA, where such thresholds denote biologically meaningful changes while minimizing false positives [48].

All raw expression matrices were quality-checked prior to analysis. Sample integrity and hybridization efficiency were verified using the Agilent Feature Extraction quality metrics. To confirm data consistency and identify potential outliers, principal component analysis (PCA) and hierarchical clustering (Euclidean distance, complete linkage) were performed using R. No samples were identified as outliers. Technical replicates and low-intensity arrays (defined as mean signal < 1.5× background) were excluded prior to normalization to ensure data reliability.

Gene expression profiling data were obtained from GEO accession GSE208143 (mRNA expression profile from RB tumors and pediatric controls [49]). The study comprised nine enucleated human RB tumors and two pediatric retina controls, each processed in biological triplicate (total *n* = 33). Total RNA was extracted using the Agilent Absolutely RNA miRNA kit, and quality was assessed via Agilent TapeStation. Twenty-five nanograms of RNA per sample were labeled with Cy3 using the Agilent Low Input Quick Amp Labeling Kit and hybridized using the one-color Agilent microarray protocol. Raw data were processed with Agilent Feature Extraction software (v11.5.1.1) and analyzed in GeneSpring GX v13.1. Differential expression was assessed using unpaired t-tests with Benjamini–Hochberg FDR correction; transcripts with FDR-adjusted *p* ≤ 0.05 and absolute fold change ≥ 2 (|log2FC| ≥ 1) were considered significant.”

For GSE208143, per-sample quality control was performed using probe intensity distributions, MA plots, and PCA clustering. No low-quality or outlier arrays were identified. The inclusion of all biological triplicates and controls was confirmed after these checks.

## 3. Results

### 3.1. Histopathological Features of Retinoblastoma and Choroidal Melanoma Subtypes

**Figure 1:**
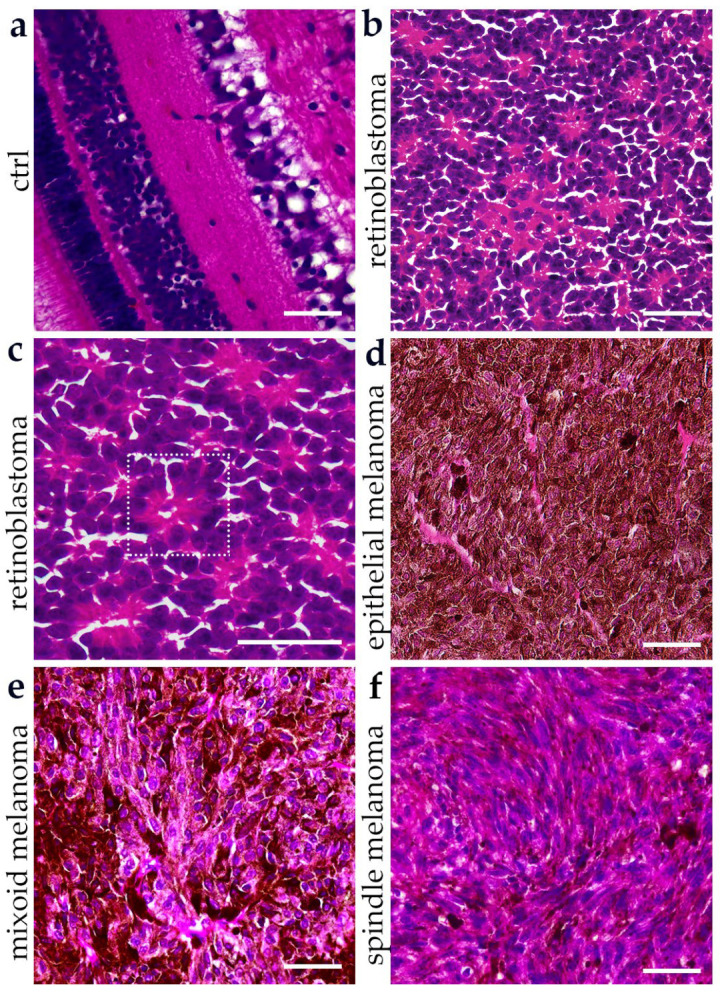
Representative hematoxylin and eosin (H&E)–stained sections of human ocular tissues. Normal retinal lamination is observed in control tissue (**a**). Retinoblastoma reveals sheets of small, round, blue cells with Flexner–Wintersteiner rosettes and Homer–Wright-type pseudorosettes (**b**,**c**). Panel (**c**) presents a higher-magnification view of the rosette structures indicated in panel b by the orange rectangle. Choroidal melanoma shows subtype-specific features: epithelioid (polygonal cells with prominent nucleoli) (**d**), spindle (elongated cells in fascicles) (**e**), and myxoid (tumor cells in a loose myxoid stroma) (**f**). Scale bars = 50 µm.

Histopathological evaluation of H&E–stained sections revealed distinct morphological features between RB and CM subtypes (Figure 1a–f). In the control retina, the expected multilayered organization was preserved, including the photoreceptor and nuclear layers (Figure 1a). RB samples demonstrated sheets of densely packed small, round cells with hyperchromatic nuclei, scant cytoplasm, and a high nuclear-to-cytoplasmic ratio, consistent with their poorly differentiated nature (Figure 1b). RB tissue demonstrates Flexner–Wintersteiner rosettes—true rosettes with central lumina indicative of photoreceptor differentiation—and occasional Homer–Wright–type pseudorosettes with central fibrillary material (Figure 1c). In contrast, CM exhibited characteristic subtype-specific architecture (Figure 1d–f). The epithelioid variant was composed of large polygonal tumor cells with abundant eosinophilic cytoplasm and prominent nucleoli (Figure 1d). The myxoid variant was distinguished by a loose stromal background with dispersed atypical melanocytes embedded in a myxoid matrix (Figure 1e). The spindle cell melanoma displayed elongated tumor cells arranged in fascicular and whorled patterns (Figure 1f).

### 3.2. LPR2 Expression Shows Focal Retention but Overall Quantitative Downregulation Across Ocular Tumor Subtypes

**Figure 2:**
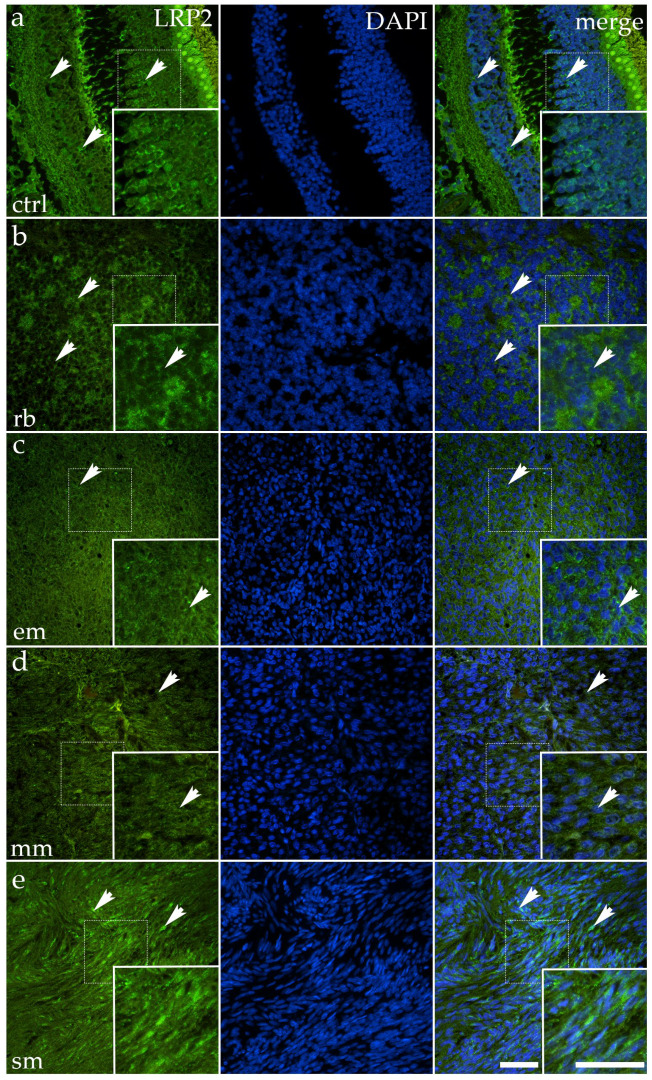
Immunofluorescence staining of LRP2, merged with 4′,6-diamidino-2-phenylindole (DAPI), in control and ocular tumors (**a**–**e**). Comparative expression of LRP2 is shown in control (ctrl; (**a**)), retinoblastoma (rb; (**b**)), epithelioid melanoma (em; (**c**)), mixoid melanoma (mm; (**d**)), and spindle melanoma (sm; (**e**)). Arrows in the LRP2 panels indicate regions of positive cytoplasmic signal. In the merged panels, arrows indicate the localization of LRP2 relative to nuclear DAPI staining. Magnification: 40×; scale bar: 50 µm.

In control ocular tissue, LPR2 immunoreactivity was modest and primarily localized to small apical epithelial clusters, producing a faint but detectable punctate cytoplasmic signal. The labeling was more subtle than expected, forming faint apical clusters rather than a continuous band. The arrows in the control panel indicate these scattered regions of positivity, where small epithelial clusters displayed slightly stronger fluorescence against the otherwise pale background (Figure 2).

Across tumor subtypes, LRP2 expression was consistently diminished. In RB, epithelioid melanoma, mixoid melanoma, and spindle melanoma, only sparse or weakly stained cells could be observed, usually in areas where remnants of epithelial organization persisted. In most regions, the fluorescent signal was extremely faint or absent altogether, and the tissue architecture appeared to be dominated by DAPI-stained nuclei. The arrows in the tumor panels highlight occasional residual foci of cytoplasmic fluorescence, underscoring the overall scarcity of Megalin expression.

**Figure 3:**
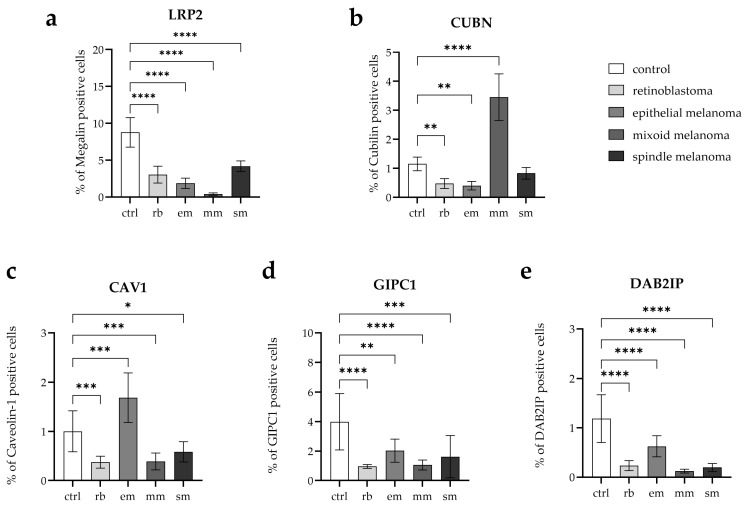
Quantitative analysis of protein expression in ocular tissues. The percentage of positively stained cells was measured for (**a**) Megalin (LRP2), (**b**) Cubilin (CUBN), (**c**) Caveolin-1 (CAV1), (**d**) GIPC1, and (**e**) DAB2IP in control tissue (ctrl), retinoblastoma (rb), epithelioid melanoma (em), mixoid melanoma (mm), and spindle melanoma (sm). Results are presented as mean ± standard deviation (SD). Statistical analysis was performed using one-way ANOVA followed by Tukey’s multiple comparison test. The following symbols indicate levels of statistical significance: * *p* < 0.05; ** *p* < 0.01; *** *p* < 0.001; **** *p* < 0.0001.

Thus, although LPR2 exhibits robust, distinct staining in isolated tumor regions, its global expression is substantially reduced when quantified across the entire tissue area. This pattern was supported by image analysis showing a significantly lower percentage of positive staining in all tumor groups compared with controls (*p* < 0.0001) (Figure 3).

These findings indicate that although baseline LPR2 expression in control ocular tissue is relatively weak, its further loss or near-complete absence in tumor tissue represents a consistent molecular change across distinct ocular malignancies. Such a pattern suggests that even low-level Megalin expression may be linked to the preservation of epithelial features, while its reduction or disappearance is associated with neoplastic transformation.

### 3.3. Cubilin Is Markedly Upregulated in Mixoid Melanoma but Reduced in Retinoblastoma and Epithelioid Melanoma

**Figure 4:**
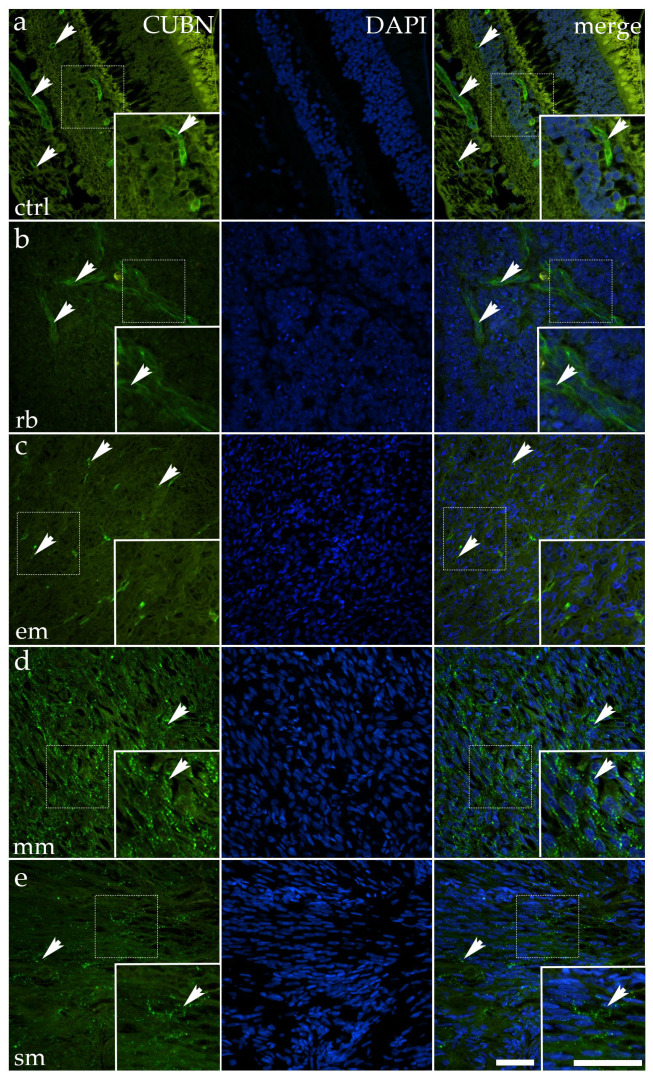
Immunofluorescence staining of Cubilin (CUBN), merged with 4′,6-diamidino-2-phenylindole (DAPI), in control and ocular tumors (**a**–**e**). Comparative expression of CUBN is shown in control (ctrl; (**a**)), retinoblastoma (rb; (**b**)), epithelioid melanoma (em; (**c**)), mixoid melanoma (mm; (**d**)), and spindle melanoma (sm; (**e**)). Arrows in the CUBN panels indicate a prominent cytoplasmic/apical signal. In merged panels, arrows show CUBN localization relative to nuclei. Magnification: 40×; scale bar: 50 µm. Occasional Cubilin positive signal is also visible in structures resembling blood vessels, consistent with known endothelial expression.

In normal ocular tissue, CUBN was clearly detectable, with green cytoplasmic fluorescence concentrated near the apical surface of epithelial cells. Although not uniformly intense, the signal followed the preserved epithelial polarity, producing a patterned distribution along the organized neuroepithelial layer. The arrows in the control panel point to these ordered cell clusters where CUBN fluorescence was most apparent. In addition to epithelial structures, isolated foci of Cubilin staining were also observed along vessel-like profiles, consistent with low-level vascular or perivascular expression (Figure 4).

In tumor tissues, the pattern varied considerably. RB showed reduced CUBN expression, with only faint, patchy fluorescent signals remaining in scattered epithelial remnants or peripheral tumor zones. The arrows in this panel mark rare foci of residual positivity within otherwise weakly stained tumor tissue. Quantitative analysis confirmed a significant reduction compared to control (*p* = 0.0038) (Figure 3).

Epithelioid melanoma also demonstrated reduced CUBN expression, characterized by weak, discontinuous fluorescence, which was limited to small epithelial clusters. Arrows highlight isolated areas of residual positivity against a vastly diminished background. Quantitative analysis confirmed a significant reduction compared to control (*p* = 0.0011) (Figure 3).

In contrast, mixoid melanoma showed a marked increase in CUBN expression. Here, the fluorescent labeling was more extensive, with broad cytoplasmic positivity that exceeded the moderate levels observed in the control. Arrows in the mixoid panel highlight these widespread, strongly positive tumor fields. This upregulation was statistically highly significant (*p* < 0.0001) (Figure 3).

Spindle melanoma displayed a heterogeneous pattern, with some regions showing preserved CUBN fluorescence, while others remained faint or negative. This variability resulted in an overall expression level similar to that of the control, with no significant difference (*p* = 0.3784) (Figure 3).

Together, these results illustrate that CUBN expression is reduced in RB and epithelioid melanoma, retained in spindle melanoma, but strikingly upregulated in mixoid melanoma, highlighting subtype-specific differences in CUBN regulation among ocular tumors.

### 3.4. CAV1 Expression Varies Between Retinoblastoma and Melanoma Subtypes

**Figure 5:**
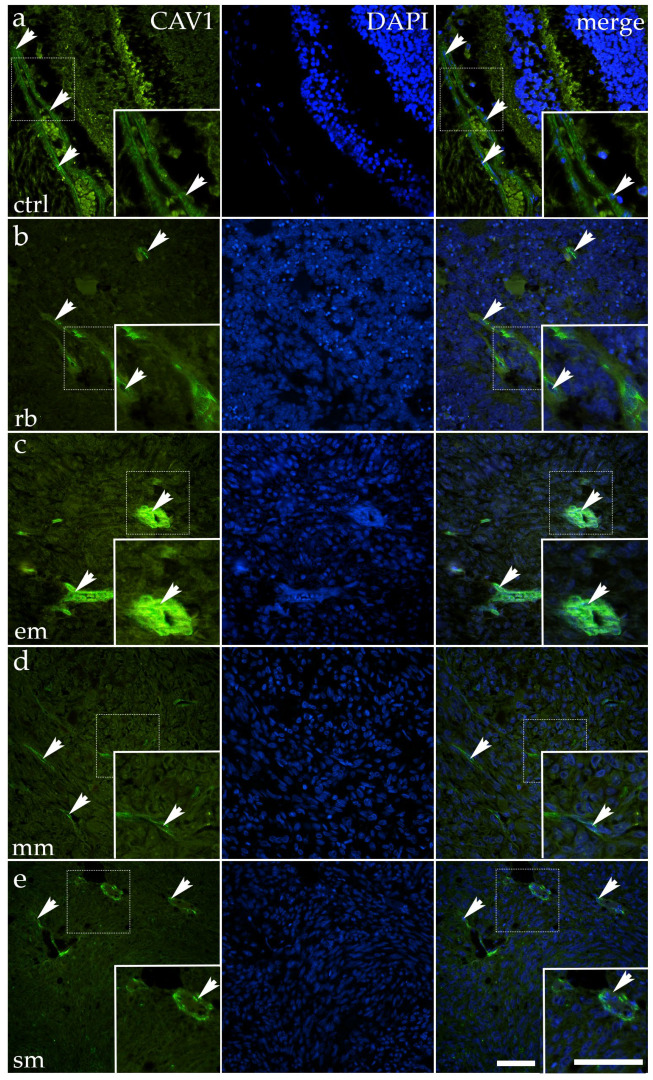
Immunofluorescence staining of Caveolin-1 (CAV1), merged with 4′,6-diamidino-2-phenylindole (DAPI), in control and ocular tumors (**a**–**e**). Comparative expression of CAV1 is shown in control (ctrl; (**a**)), retinoblastoma (rb; (**b**)), epithelioid melanoma (em; (**c**)), mixoid melanoma (mm; (**d**)), and spindle melanoma (sm; (**e**)). Arrows in the CAV1 panels indicate regions of positive cytoplasmic and membranous signals. In merged panels, arrows indicate CAV1 localization in relation to nuclear DAPI staining. Magnification: 40×; scale bar: 50 µm. Occasional Caveolin-1 positive signal is also visible in structures resembling blood vessels, consistent with known endothelial expression.

In normal ocular tissue, CAV1 was moderately expressed, with green cytoplasmic fluorescence most apparent in the epithelial compartment. The signal outlined epithelial cells in an organized fashion, though the intensity was not uniformly strong. The arrows in the control panel highlight clusters where the fluorescence was concentrated along the epithelial lining, reflecting preserved polarity. Occasional Caveolin-1–positive signals were also present in structures resembling blood vessels, which is expected given the known endothelial expression of caveolar proteins (Figure 5).

RB showed a marked reduction in CAV1 expression. Only occasional, faintly fluorescent cells were visible against a background of densely nucleated tumor tissue. Arrows indicate these rare residual foci. Quantitative analysis confirmed significantly lower expression compared to control (*p* = 0.0006) (Figure 3).

Mixoid melanoma also displayed reduced CAV1, with patchy, weakly positive fluorescent areas scattered irregularly throughout the tumor mass. Arrows highlight these sparse signals, contrasting with wide regions lacking stain. Quantitative analysis confirmed a significant decrease compared to control (*p* = 0.0008) (Figure 3).

Spindle melanoma exhibited a heterogeneous pattern, with some elongated cells retaining moderate cytoplasmic positivity, while large areas were nearly negative. Arrows emphasize preserved tracts of fluorescence within otherwise weakly stained fields. Quantitative analysis revealed a significant reduction compared to the control (*p* = 0.0336) (Figure 3).

In contrast, epithelioid melanoma showed an evident upregulation of CAV1. The fluorescent signal was more substantial and more widespread than in the control, extending across tumor nests. Arrows highlight regions where cytoplasmic fluorescence was particularly dense and uniform. Quantitative analysis confirmed a significant increase compared to control (*p* = 0.002) (Figure 3).

Taken together, CAV1 expression is downregulated in RB, mixoid melanoma, and spindle melanoma, but upregulated in epithelioid melanoma, suggesting divergent roles for this protein across ocular tumor subtypes.

### 3.5. GIPC-1 Expression Is Significantly Reduced in All Ocular Tumor Subtypes

**Figure 6:**
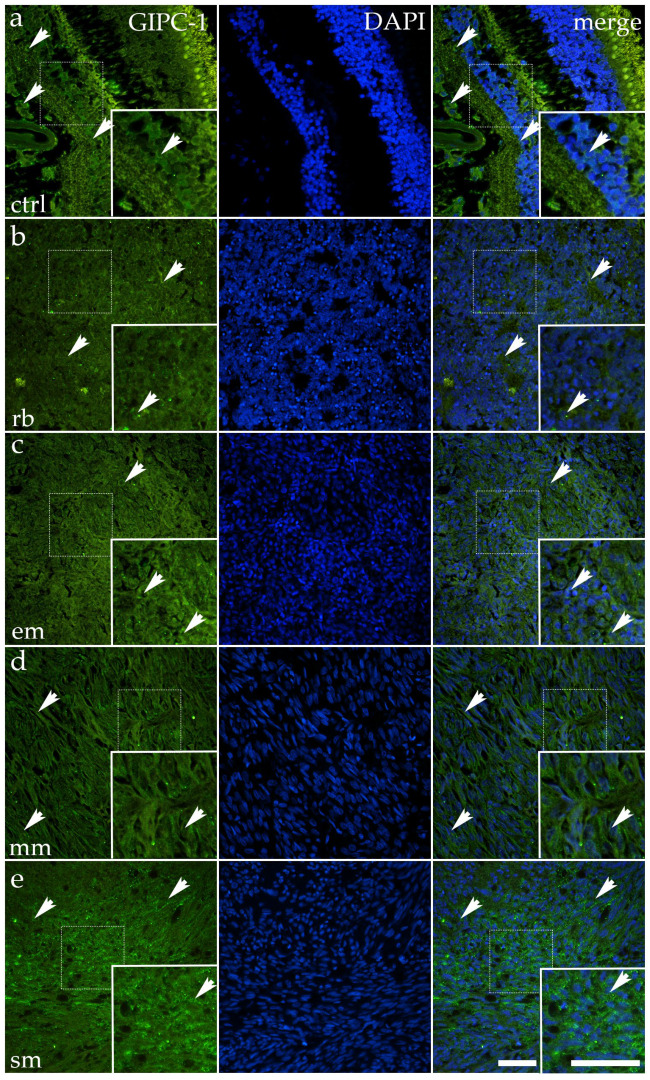
Immunofluorescence staining of GIPC-1, merged with 4′,6-diamidino-2-phenylindole (DAPI), in control and ocular tumors (**a**–**e**). Comparative expression of GIPC-1 is shown in control (ctrl; (**a**)), retinoblastoma (rb; (**b**)), epithelioid melanoma (em; (**c**)), mixoid melanoma (mm; (**d**)), and spindle melanoma (sm; (**e**)). Arrows in the GIPC-1 panels mark areas of notable cytoplasmic signal. In merged panels, arrows show the spatial relationship between GIPC-1 and nuclei. Magnification: 40×; scale bar: 50 µm.

In normal ocular tissue, GIPC-1 was moderately expressed in the epithelium, where green cytoplasmic fluorescence appeared as discrete puncta aligned with the apical cytoplasm. The arrows in the control panel indicate these organized clusters, in which expression was preserved alongside epithelial architecture (Figure 6).

RB displayed markedly reduced GIPC-1 expression. Only scattered, faint cytoplasmic fluorescence was detectable in small clusters of cells. The arrows point to these weak residual signals. Quantitative analysis confirmed significantly lower expression compared to control (*p* < 0.0001) (Figure 3).

Mixoid melanoma also showed reduced GIPC-1, with fragmented, faint fluorescence confined to small patches. Arrows highlight these discontinuous regions of the signal. Quantitative analysis confirmed a significant decrease compared to control (*p* < 0.0001) (Figure 3).

Spindle melanoma retained more GIPC-1 than mixoid melanoma, but overall staining remained weaker than in the control. Expression appeared linear in some elongated cells, while large areas lacked detectable fluorescence. Arrows mark surviving tracts of positivity. Quantitative analysis confirmed significantly reduced expression compared to control (*p* = 0.0003) (Figure 3).

In contrast, epithelioid melanoma largely resembled the control pattern. Tumor nests exhibited moderate cytoplasmic fluorescence across epithelial-like clusters, as highlighted by arrows. Statistical analysis showed a significant difference compared to the control (*p* = 0.0032) (Figure 3).

Altogether, GIPC-1 expression is well preserved in control and epithelioid melanoma but significantly downregulated in RB, mixoid melanoma, and spindle melanoma, reflecting subtype-specific differences in the maintenance of epithelial adaptor proteins.

### 3.6. DAB2IP Expression Is Retained in Epithelioid Melanoma but Diminished in Retinoblastoma, Mixoid Melanoma, and Spindle Melanoma

**Figure 7:**
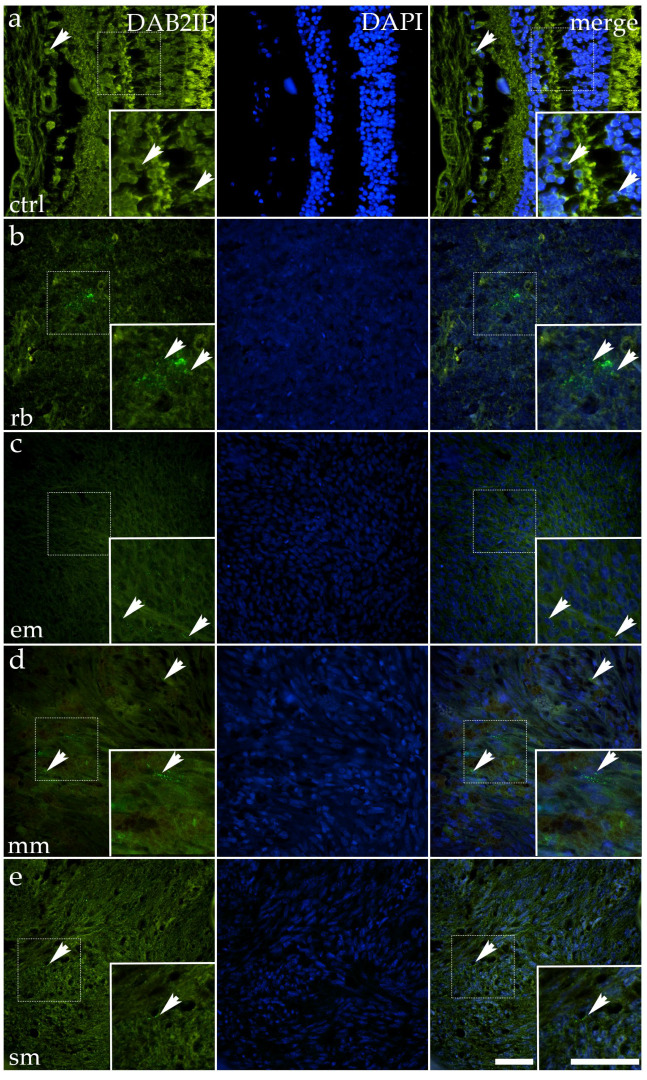
Immunofluorescence staining of DAB2IP, merged with 4′,6-diamidino-2-phenylindole (DAPI), in control and ocular tumors (**a**–**e**). Comparative expression of DAB2IP is shown in control (ctrl; (**a**)), retinoblastoma (rb; (**b**)), epithelioid melanoma (em; (**c**)), mixoid melanoma (mm; (**d**)), and spindle melanoma (sm; (**e**)). Arrows in the DAB2IP panels indicate regions of positive perinuclear/cytoplasmic staining. In merged panels, arrows indicate DAB2IP localization relative to nuclear DAPI staining. Magnification: 40×; scale bar: 50 µm.

In control ocular tissue, DAB2IP was moderately expressed, with green cytoplasmic fluorescence outlining epithelial cells in an orderly fashion. The signal appeared granular and perinuclear, producing a distinct contrast with surrounding nuclei. The arrows in the control panel highlight these compact epithelial rows, where the distribution of DAB2IP mirrored the preserved architecture (Figure 7).

RB showed reduced DAB2IP expression. The fluorescent signal appeared faint and patchy, confined to occasional cells scattered within densely nucleated tumor regions. Arrows indicate these sparse residual foci. Quantitative analysis revealed significantly lower expression compared to control (*p* < 0.0001) (Figure 3).

In epithelioid melanoma, DAB2IP expression was largely comparable to that of the control. Tumor nests retained cytoplasmic fluorescence of moderate intensity, and the arrows mark preserved epithelial-like clusters. Statistical analysis confirmed no significant difference compared to the control.

Mixoid melanoma exhibited a marked loss of DAB2IP. Only rare, weakly fluorescent cells were visible, highlighted by arrows in the tumor panel. Quantitative analysis confirmed a dramatic reduction compared to control (*p* < 0.0001) (Figure 3).

Spindle melanoma showed intermediate expression. Some elongated tumor cells retained moderate fluorescence, but most regions appeared weak or negative. Arrows emphasize preserved tracts of signal within otherwise diminished tissue. Quantitative analysis confirmed significantly lower expression compared to control (*p* < 0.0001) (Figure 3).

Altogether, DAB2IP is robustly expressed in control tissue and retained in epithelioid melanoma, but significantly downregulated in RB, mixoid melanoma, and spindle melanoma, suggesting that loss of this tumor suppressor may accompany neoplastic progression in specific ocular malignancies.

### 3.7. Survival Analyses

To evaluate the prognostic relevance of *LRP2*, *CUBN*, *DAB2IP*, *GIPC1*, and *CAV1* in uveal melanoma, we performed overall survival analysis using the GEPIA2 online platform. The analysis was based on gene expression data from the TCGA Uveal Melanoma (UVM) cohort (*n* = 78 patients with survival data). Patients were stratified into high- and low-expression groups using the median expression as the cutoff, and survival differences were assessed using the Log-rank test.

**Figure 8:**
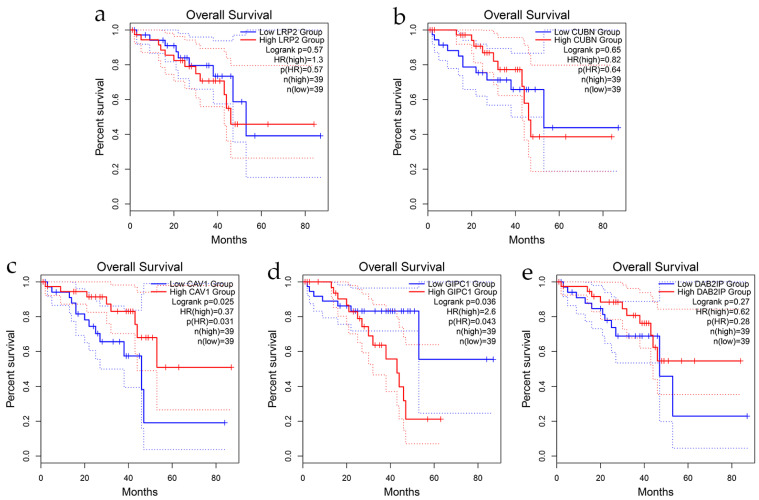
Kaplan–Meier survival curves showing the association between overall survival and the expression levels of *Megalin* (*LRP2*) (**a**), *Cubilin* (*CUBN*) (**b**), *Caveolin 1* (*CAV1*) (**c**), *Disabled homolog 2-interacting protein* (DAB2IP) (**d**), and *GIPC PDZ domain-containing protein 1* (*GIPC1*) (**e**), in uveal melanoma patients. The analysis was conducted using the publicly available GEPIA2 database (http://gepia2.cancer-pku.cn/, accessed 30 July 2025), based on data from The Cancer Genome Atlas (TCGA) Ocular Melanoma (UVM) cohort. Patients were dichotomized into high- and low-expression groups using the median expression value as the cutoff. Solid lines represent survival probability, dotted lines indicate the 95% confidence intervals, and vertical tick marks denote censored cases (patients alive at last follow-up or lost to follow-up).

Kaplan–Meier survival curves showed that high expression of CAV1 and GIPC1 was significantly associated with worse overall survival (CAV1: HR = 3.0, 95% CI 1.11–8.10, *p* = 0.025; GIPC1: HR = 2.6, 95% CI 1.03–6.56, *p* = 0.036) suggesting a potential oncogenic or tumor-promoting role of these genes in UVM (Figure 8). Given the modest sample size of the TCGA-UVM cohort (*n* = 78), confidence intervals are relatively wide, particularly for CAV1 and GIPC1, reflecting uncertainty in effect size estimates. To assess independent prognostic value while adjusting for potential confounders, multivariate Cox proportional hazards regression was subsequently performed (Section 3.9, Figure 10).

In contrast, expression levels of LRP2 (HR = 1.3, 95% CI 0.72–2.35, *p* = 0.57), CUBN (HR = 0.82, 95% CI 0.47–1.43, *p* = 0.65), and DAB2IP (HR = 1.2, 95% CI 0.68–2.12, *p* = 0.27) were not significantly associated with overall survival (Figure 8).

### 3.8. Differential Gene Expression Analysis

**Figure 9:**
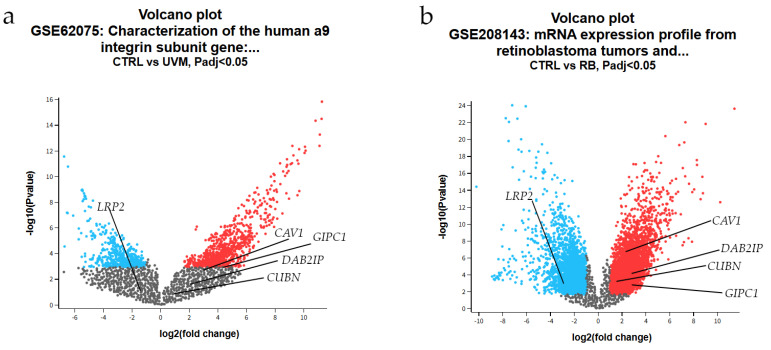
Volcano plots showing differential gene expression in two transcriptomic datasets. Differential expression analysis comparing primary human uveal melanocyte cultures (CTRL) and three uveal melanoma cell lines (T115, T142, T143; UVM) from dataset GSE62075 (**a**). Differential expression analysis comparing retinoblastoma tumors (RB) and pediatric control retinae (CTRL) from dataset GSE208143 (**b**). The x-axis represents log2(fold change), and the y-axis represents –log10(adjusted *p*-value). Each dot represents a gene. Genes with an adjusted *p*-value less than 0.05 (−log10 > 1.3) are considered significantly differentially expressed and are shown in color: red for upregulated and blue for downregulated genes. Non-significant genes are shown in gray. The positions of *Megalin* (*LRP2*), *Cubilin* (*CUBN*), *Caveolin 1* (*CAV1*), *GIPC PDZ domain containing family*, *member 1* (*GIPC1*), and *Disabled homolog 2-interacting protein* (*DAB2IP*) are annotated. In GSE62075, none of these genes were significantly differentially expressed between CTRL and UVM samples. In contrast, in GSE208143, *LRP2* was significantly downregulated in RB compared to controls, while *CUBN*, *CAV1, GIPC1,* and *DAB2IP* were significantly upregulated in RB tissue.

Differential expression analysis was performed to compare ocular tumor samples with their respective controls in two independent transcriptomic datasets (Figure 9). In dataset GSE62075, which included primary human uveal melanocyte cultures and three uveal melanoma cell lines (T115, T142, T143), global transcriptional differences were observed; however, none of the selected genes of interest (*LRP2*, *CUBN*, *CAV1, DAB2IP*, and *GIPC1*) reached statistical significance (Figure 9a). In contrast, analysis of dataset GSE208143, comprising RB tumors and pediatric control retinae, revealed marked differential expressions. Specifically, *LRP2* was significantly downregulated in RB compared to controls, whereas *CUBN*, DAB2*IP*, *GIPC1*, and *CAV1* were significantly upregulated in tumor tissue (Figure 9b).

### 3.9. Multivariate Cox Regression Analysis of Gene Expression and Survival in Uveal Melanoma

**Figure 10:**
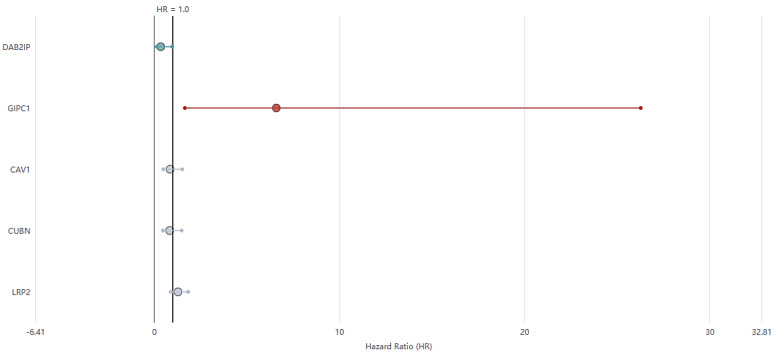
Multivariate Cox proportional hazards regression analysis of candidate genes in uveal melanoma (TCGA-UVM cohort). Forest plot generated using the kircv3 online platform, showing the hazard ratios (HRs) with 95% confidence intervals (CIs) for the expression of *LRP2*, *CUBN*, *DAB2IP*, *GIPC1*, and *CAV1* in relation to overall survival. High expression of *DAB2IP* was associated with a significantly reduced risk of mortality (protective factor), whereas high expression of *GIPC1* was significantly associated with increased mortality risk (adverse prognostic factor). The remaining genes (*CAV1*, *CUBN*, and *LRP2*) did not reach statistical significance.

To assess the prognostic significance of selected genes in uveal melanoma, a multivariate Cox proportional hazards regression analysis was performed. Among the five genes evaluated, *DAB2IP* and *GIPC1* demonstrated statistically significant associations with overall survival (Figure 10). *DAB2IP* expression was associated with a favorable prognosis, exhibiting a hazard ratio (HR) of 0.348 (95% confidence interval (CI): 0.126–0.962, *p* = 0.042), indicating that elevated expression levels were significantly correlated with reduced risk of mortality. Conversely, *GIPC1* was identified as a potential risk factor, with high expression associated with significantly poorer survival outcomes (HR = 6.591, 95% CI: 1.653–26.271, *p* = 0.00752). The remaining genes—*CAV1* (HR = 0.854, 95% CI: 0.483–1.509, *p* = 0.587), *CUBN* (HR = 0.833, 95% CI: 0.471–1.476, *p* = 0.532), and *LRP2* (HR = 1.278, 95% CI: 0.894–1.826, *p* = 0.179)—did not reach statistical significance (Figure 10).

## 4. Discussion

### 4.1. Overview

The pathogenesis of ocular tumors involves alterations in endocytosis, nutrient uptake, and intracellular signaling [8]. LRP2 and CUBN are multiligand endocytic receptors essential for receptor-mediated transport [9,10]. CAV1 is the caveolar scaffolding protein with roles in retinal physiology and ocular disease [19,20,21]. GIPC1 is an adaptor linking receptors to PI3K–AKT and RhoA pathways, coordinating trafficking and motility [25,26,27]. DAB2IP is a tumor suppressor that restrains ERK/AKT/NF-κB signaling and EMT across cancers [31,32,33]. However, expression patterns of these proteins in human RB and CM remain under-characterized. Here, we systematically integrate immunofluorescence, public transcriptomics, and survival analyses to delineate their subtype-linked expression in ocular tumors.

### 4.2. LRP2/Megalin Downregulation in Ocular Tumors

In our cohort, LRP2 showed only weak baseline immunofluorescence in control ocular tissue and was uniformly downregulated across RB and all CM subtypes, indicating that loss of LRP2 accompanies ocular tumorigenesis. Because subtype-resolved ocular data for LRP2 remain sparse, related evidence from development and other tumor types helps contextualize our results.

Rasmussen et al. reported that across TCGA, tumors arising from LRP2-positive epithelia frequently silence the receptor. Lower LRP2 was associated with dedifferentiated subtypes and poorer survival in clear-cell/papillary renal cell carcinoma, papillary thyroid carcinoma, mesothelioma, and invasive breast carcinoma, with methylation-linked repression as a plausible mechanism [8]. Our results are consistent with this pattern, as ocular tumors also showed LRP2 loss in association with dedifferentiation.

Although not directly studied in large ocular melanoma cohorts, research on cutaneous melanoma suggests that tumors can aberrantly acquire LRP2 and depend on it for proliferation and survival. By contrast, cancers without a strong baseline epithelial LRP2 can aberrantly gain Megalin and exploit its functions. Andersen et al. showed that more than 60% of malignant cutaneous melanomas (compared to ~20% of nevi) express LRP2, and that *LRP2* knockdown decreases melanoma cell proliferation and survival, underscoring a growth-supporting role in this context [50]. Our data diverge from this cutaneous pattern: in ocular melanoma, we observed consistent downregulation rather than acquisition of LRP2, indicating tissue-specific differences in LRP2′s role along the melanocytic lineage.

Functionally, LRP2 mediates endocytosis of diverse ligands (retinoids, vitamin D complexes, lipoproteins, hormones) and modulates morphogen signaling. Christ et al. demonstrated that in the developing eye, LRP2 limits Sonic Hedgehog (SHH) activity by ligand clearance at the retinal margin; loss of *LRP2* leads to ectopic SHH signaling and hyperproliferation, revealing a growth-restraining function in ocular tissues [14]. This mechanism aligns with our findings, as uniform LRP2 expression loss in RB and CM is compatible with the removal of growth control and impaired uptake of differentiation-promoting micronutrients (retinoids/vitamin D).

Although arising outside the eye, colon cancer data further underscore the tissue-specificity of LRP2′s role in tumor biology. Extending this context dependence beyond melanoma, Zhang et al. concluded that in colon adenocarcinoma, higher LRP2 expression correlates with worse prognosis and higher pathological stage, and that forced LRP2 overexpression increases proliferation in COAD cell lines, again illustrating that tumors can either lose LRP2 (with dedifferentiation) or co-opt it to support growth, depending on tissue context-a pattern contrasting with our ocular tumor data [51]. Taken together, these findings indicate that LRP2 behaves in a tumor-suppressor-like, differentiation-linked manner in ocular tumors (consistent with our downregulation). In contrast, in other tissues (e.g., skin, colon), it can be co-opted to support growth, underscoring strong tumor-type specificity. In this framework, our findings position LRP2 loss as a reproducible molecular feature of ocular tumors, motivating future functional studies to test whether restoring megalin-dependent nutrient/morphogen handling or restraining SHH-pathway activity could mitigate the proliferative advantage conferred by its absence.

The mechanistic consequences of LRP2 loss extend beyond morphogen dysregulation to fundamentally alter cellular nutrient homeostasis. LRP2-mediated endocytosis normally delivers retinoids and vitamin D metabolites—both critical regulators of epithelial differentiation and cell cycle exit—to intracellular compartments where they activate nuclear receptors [52,53,54]. Specifically, megalin facilitates the uptake of vitamin A-binding proteins and vitamin D-binding protein (DBP) complexes, enabling intracellular accumulation of these lipophilic vitamins [52,53]. Once internalized, these ligands bind to the vitamin D receptor (VDR) and retinoic acid receptors (RAR/RXR), which translocate to the nucleus and directly activate transcription of cyclin-dependent kinase inhibitors p21WAF1/CIP1 and p27KIP1 [55,56,57]. These CDK inhibitors arrest cells in G1 phase, blocking progression to S phase and promoting differentiation [57,58]. In ocular tumors, uniform LRP2 downregulation would therefore create a dual proliferative advantage: first, by removing SHH pathway restraint (as demonstrated by Christensen et al.), and second, by depriving cells of differentiation signals ordinarily transduced through vitamin A and D-dependent transcriptional programs [56,57,58]. This convergence of impaired morphogen clearance and reduced micronutrient delivery may explain why LRP2 loss is uniformly observed across both RB and CM subtypes despite their distinct cellular origins.

### 4.3. Subtype-Specific CUBN Expression Patterns

Across this series, CUBN showed distinct, subtype-specific expression patterns. Because ocular, subtype-resolved data for CUBN are limited, we interpret these patterns in conjunction with established mechanistic roles in nutrient uptake and vitamin metabolism. In normal ocular tissue, CUBN was present along the epithelial lining. In RB and epithelioid melanoma, CUBN expression was markedly reduced, while spindle melanoma retained expression at control-like levels. Notably, myxoid melanomas demonstrated upregulation of CUBN, exceeding normal tissue.

Gremel et al. demonstrated that the CUBN protein was expressed in more than half of clear cell renal carcinomas, and that its loss correlated with a worse prognosis, independent of tumor stage and grade [13]. Our data parallel this observation, as CUBN downregulation in RB and epithelioid melanoma may similarly reflect dedifferentiation and a more aggressive phenotype. Supporting this, Niinivirta et al. demonstrated that CUBN positivity predicted better progression-free and overall survival in metastatic RCC patients treated with sunitinib or sorafenib [17], underscoring CUBN as a marker of favorable biology when retained.

By contrast, Wu et al. reported that *CUBN* mRNA was overexpressed in colorectal cancer, where high expression correlated with advanced stage and shorter survival [18]. Similarly, our finding of CUBN upregulation in myxoid melanomas mirrors this pattern, suggesting that in specific tumor contexts, CUBN may be co-opted to promote growth or altered nutrient handling. Epigenetic control adds further complexity: Aseem et al. showed that CUBN is subject to monoallelic expression and is positively regulated by PPARα/γ, with DNA methylation and histone deacetylation suppressing its transcription [59]. This evidence provides a mechanistic framework whereby *CUBN* loss in some ocular tumors could reflect epigenetic silencing.

Functionally, CUBN is a multiligand receptor for vitamin B12, vitamin D–binding protein, lipoproteins, and iron complexes. CUBN downregulation in aggressive tumors may represent a metabolic shift from receptor-mediated nutrient uptake toward alternative mechanisms supporting rapid proliferation. Loss of CUBN-mediated vitamin B12 uptake impairs one-carbon metabolism essential for nucleotide synthesis [60,61,62,63,64,65]. CUBN facilitates endocytosis of intrinsic factor-B12 complexes; intracellularly, B12 serves as cofactor for methionine synthase, catalyzing homocysteine to methionine conversion—critical for DNA methylation and purine/pyrimidine biosynthesis [62,63]. Reduced vitamin D-binding protein uptake diminishes VDR-dependent p21/p27 transcription, enabling proliferation [13,17,56,65]. Given the sparse ocular literature, we refrain from broad generalization and restrict our inference to the analyzed subtypes.

Taken together, these findings establish that CUBN is downregulated in aggressive ocular tumor types but retained or upregulated in less aggressive or mixoid forms, suggesting a role as a marker of differentiation state and a potential mediator of tumor metabolism in ocular oncology.

### 4.4. Context-Dependent CAV1 Expression Across Melanoma Subtypes

Across this series, CAV1 showed subtype-specific expression patterns. Because subtype-resolved ocular data for CAV1 are limited, we interpret these patterns in conjunction with established caveolae biology and ocular physiology. CAV1 protein was downregulated in RB, spindle melanoma, and myxoid melanoma, but upregulated in epithelioid melanoma compared with normal ocular tissue. This suggests that CAV1 loss may be associated with less aggressive or more differentiated tumors, whereas its induction is characteristic of aggressive phenotypes, such as epithelioid melanoma.

Stenzel et al. analyzed CAV1 in 51 uveal melanomas and found it broadly expressed, with particularly high levels in large, metastasis-prone tumors [24]. Our results parallel this observation, as epithelioid melanomas–the most aggressive subtype–also showed increased CAV1 protein, supporting the link between Cav-1 and tumor progression in uveal melanoma.

By contrast, Doddi et al. reported that *CAV1* mRNA was significantly downregulated in metastatic uveal melanoma compared with non-metastatic cases [66]. This diverges from our protein-level data, which showed upregulation in aggressive epithelioid tumors. This discrepancy highlights the possibility of post-transcriptional or post-translational regulation and underscores the need to assess both transcript and protein levels to understand Cav-1 biology in ocular tumors.

Belkot et al. demonstrated that uveal melanoma cell lines expressed markedly lower CAV1 compared with cutaneous melanoma lines [67]. This finding is consistent with our observations in spindle and myxoid melanomas, where CAV1 expression was reduced, suggesting that the downregulation of Cav-1 is a characteristic feature of specific ocular tumor subtypes and may distinguish them from cutaneous melanoma biology.

Taskaeva et al. found that uveal melanoma cells contained a higher density of caveolae, plasma membrane structures that depend on CAV1, compared with normal choroidal melanocytes [68]. This supports our finding of CAV1 upregulation in epithelioid melanoma, as the increased caveolae in UM imply functional Cav-1 involvement in tumor progression.

Finally, Lobos-González et al. showed in murine melanoma models that Cav-1 overexpression reduced primary tumor growth but enhanced metastatic spread [22]. This dual role aligns with our findings: the loss of CAV1 in RB and spindle/myxoid melanoma suggests a tumor-suppressor role in less aggressive settings, while its gain in epithelioid melanoma points to its pro-metastatic activity.

Taken together, these findings indicate that CAV1 acts as a context-dependent regulator in ocular tumors: it is reduced in RB and less aggressive melanomas, but induced in epithelioid melanoma, reflecting its dual roles in tumor suppression and metastasis promotion.

### 4.5. GIPC1 Downregulation and Receptor Trafficking in Ocular Tumor

Across this series, GIPC1 showed subtype-specific expression patterns. Because subtype-resolved ocular data for GIPC1 are limited, we interpret these findings in conjunction with its established roles in receptor trafficking and signaling. In normal ocular tissue, GIPC1 was moderately expressed along epithelial structures. In RB, spindle melanoma, and myxoid melanoma, GIPC1 protein levels were markedly reduced, while epithelioid melanoma showed no significant difference compared with control tissue.

Katoh et al. reported that GIPC1 is frequently overexpressed in breast, ovarian, and pancreatic cancers, where it promotes proliferation and invasion. In contrast, in specific contexts, such as HPV-positive cervical cancer, GIPC1 downregulation confers resistance to growth-suppressive signals [69]. Our results differ from the high-expression profile observed in many carcinomas, as ocular tumors more commonly exhibit reduced GIPC1 expression, suggesting that GIPC1 loss may accompany dedifferentiation in RB and spindle melanoma.

Li et al. demonstrated that GIPC1 overexpression in gastric cancer activates PDGFR-driven PI3K–AKT signaling, enhancing tumor cell proliferation and migration [27]. In contrast, our study reveals that ocular tumors rarely exhibit GIPC1 overexpression, with epithelioid melanoma maintaining baseline levels rather than increasing expression. This indicates that GIPC1-driven PI3K–AKT signaling may be less central in ocular malignancies than in gastric or breast cancer.

Bao et al. demonstrated that neuropilin-1 stimulates a GIPC1–Syx complex, which activates RhoA and degrades p27^Kip1, thereby promoting tumor cell proliferation and migration [30]. By comparison, our findings suggest that ocular tumors with GIPC1 downregulation may lack this signaling axis, possibly reducing invasive potential relative to tumors that maintain higher levels of GIPC1.

Siegel et al. demonstrated that GIPC1 acts as both a binding partner and a transcriptional regulator of MACC1, thereby enhancing the metastatic potential of colorectal cancer [70]. By contrast, our data in ocular tumors show predominant GIPC1 downregulation, suggesting that GIPC1-driven pro-metastatic signaling may not be a dominant mechanism in the ocular tumor microenvironment. Given the sparse ocular literature by subtype, we refrain from broad generalization and restrict our inference to the analyzed contexts.

Taken together, these findings indicate that GIPC1 is downregulated in RB, spindle, and myxoid melanoma, while epithelioid melanoma retains control-like expression. Unlike in other cancers, where GIPC1 overexpression promotes aggressiveness, ocular tumors exhibit a distinct pattern, suggesting that GIPC1 loss may be linked to dedifferentiation but not necessarily to progression.

### 4.6. DAB2IP as a Tumor Suppressor in Ocular Malignancies

In our analysis, DAB2IP showed subtype-specific expression patterns. Because subtype-resolved ocular data for DAB2IP are limited, we interpret these findings alongside its established tumor-suppressor functions. DAB2IP protein was significantly downregulated in RB, spindle melanoma, and myxoid melanoma compared with normal ocular tissue, while no significant difference was observed in epithelioid melanoma. This suggests that loss of DAB2IP is linked to dedifferentiated tumor types, whereas epithelioid melanoma maintains baseline expression despite its aggressive phenotype.

Sun et al. reported that DAB2IP knockdown in gastric cancer cells enhanced proliferation, migration, and epithelial–mesenchymal transition (EMT) through activation of the ERK pathway [33]. This finding aligns with our observations in spindle and myxoid melanomas, where DAB2IP loss may drive EMT-like features and invasiveness.

Similarly, Wu et al. showed that re-expression of DAB2IP in colorectal cancer suppressed proliferation and migration, reduced EMT markers, and inhibited both AKT and ERK phosphorylation [71]. Our data on RB are consistent with this mechanism, as reduced DAB2IP may relieve the inhibition of PI3K–AKT/ERK signaling, thereby supporting tumor growth.

Smits et al. demonstrated that EZH2-mediated histone methylation silences DAB2IP in medulloblastoma, contributing to apoptosis resistance and poorer outcomes [72]. This provides a plausible explanation for our RB findings, as both tumors share developmental origins in neural progenitors and may undergo similar epigenetic repression of DAB2IP.

Bellazzo et al. identified microRNAs, such as miR-149-3p, that directly target DAB2IP, activating NF-κB signaling and enhancing invasion [73]. This mechanism could underlie the marked downregulation we observed in spindle and myxoid melanomas, while epithelioid melanoma may escape such microRNA-mediated silencing, explaining its preserved expression despite aggressiveness.

Zhang et al. demonstrated that DAB2IP downregulates HSP90AA1, thereby inhibiting malignant behaviors in colorectal cancer. They showed that DAB2IP loss promoted proliferation, migration, and resistance to apoptosis, while re-expression of DAB2IP counteracted these features through the HSP90AA1/SRP9/ASK1/JNK axis [74]. These findings parallel our results: in ocular tumors, reduced DAB2IP may similarly relieve inhibition of survival signaling, facilitating tumor progression.

Mechanistically, DAB2IP loss creates a permissive signaling environment through coordinated pathway activation [75,76,77]. As a RAS-GAP, DAB2IP normally promotes RAS-GTP hydrolysis; its loss sustains RAS-GTP loading, driving constitutive ERK1/2 phosphorylation and proliferative gene expression [75,76]. Simultaneously, loss of DAB2IP inhibition of PI3K (via direct p85 binding) allows unrestrained AKT activation, suppressing pro-apoptotic BAD/FOXO while activating mTOR [77,78,79]. ERK and AKT hyperactivation converge with NF-κB signaling (normally inhibited by DAB2IP) to create a self-reinforcing loop [76,80]. NF-κB transcribes survival genes (BCL-2, BCL-XL, survivin, c-IAPs, XIAP) that block both extrinsic (caspase-8 inhibition) and intrinsic (mitochondrial cytochrome c release prevention) apoptotic pathways [80,81,82,83]. This explains why DAB2IP loss simultaneously enhances proliferation (ERK), promotes survival (AKT/mTOR), and confers treatment resistance (NF-κB).

Taken together, DAB2IP was significantly reduced in RB, spindle, and myxoid melanoma, but not in epithelioid melanoma, suggesting that DAB2IP loss is more pronounced in more dedifferentiated histotypes. This pattern aligns with DAB2IP’s tumor-suppressor role, where its repression (epigenetic/microRNA) unleashes ERK/AKT/NF-κB signaling, proliferation, EMT, and resistance to apoptosis, while preserved expression in epithelioid melanoma suggests a reliance on alternative oncogenic programs.

### 4.7. Conceptual Overview

To provide additional conceptual clarity, we include here a synthesized overview that integrates the molecular pathways with the histopathological behavior of ocular tumors. LRP2 and CUBN, as key endocytic nutrient receptors, appear linked to differentiation status and micronutrient uptake, with their downregulation in aggressive histotypes suggesting impaired ligand handling and deregulated morphogen signaling. Caveolin-1 demonstrates context-dependent roles, acting as a tumor suppressor in RB and less aggressive melanomas, but supporting invasive features in epithelioid melanoma. GIPC1 and DAB2IP, which regulate PI3K–AKT, ERK, and RhoA pathways, are predominantly reduced in dedifferentiated tumor subtypes, consistent with the release of growth-promoting signaling. Together, these coordinated alterations in endocytosis, membrane organization, nutrient acquisition, and intracellular signaling outline a unified molecular framework that aligns with the histopathological patterns observed across ocular tumor subtypes. Beyond their biological relevance, the subtype-specific protein patterns identified here have clear translational potential. Incorporating LRP2, CUBN, Caveolin-1, GIPC1, and DAB2IP into biomarker-driven clinical trial design could enhance risk stratification in uveal melanoma and RB and support the development of prognostic signatures that distinguish aggressive from indolent disease. These markers may aid in defining biomarker-enriched trial cohorts, improving patient selection, and guiding evaluation of pathway-targeted therapies, ultimately contributing to more individualized surveillance and treatment strategies in ocular oncology.

### 4.8. Prognostic Associations and Transcriptomic Validation

Kaplan–Meier survival analysis showed that high expression of *CAV1* and *GIPC1* was associated with worse overall survival in uveal melanoma, whereas *LRP2*, *CUBN*, and *DAB2IP* did not reach significance. The prognostic associations we observed must be interpreted within the context of the TCGA-UVM cohort size (*n* = 80 patients). While *CAV1* and *GIPC1* reached statistical significance in univariate analysis, the modest sample size limits statistical power and is reflected in the relatively wide confidence intervals. These findings are hypothesis-generating and require validation in larger, independent cohorts before clinical implementation. The loss of *CAV1*′s prognostic significance in multivariate models despite univariate significance suggests that its effects may be mediated through or confounded by other clinical-pathological variables, highlighting the importance of accounting for multiple covariates in survival analyses. By contrast, *GIPC1* retained independent prognostic significance even after multivariate adjustment, though the wide confidence interval reflects substantial uncertainty and mandates external validation.

To assess reproducibility and generalizability, we interrogated multiple independent transcriptomic datasets. Our protein-level observation of *LRP2* downregulation in ocular tumors was directly corroborated at the mRNA level in GSE208143 (RB vs. control retina), where *LRP2* transcripts were significantly reduced. This cross-platform validation (protein immunofluorescence vs. mRNA expression) strengthens confidence in *LRP2* loss as a consistent, reproducible molecular feature of RB. However, notable protein-mRNA discordances emerged for *CUBN*, *CAV1*, *DAB2IP*, and *GIPC1*, which showed transcriptional upregulation in GSE208143 despite protein-level downregulation in most tumor subtypes. These discordances likely reflect post-transcriptional regulatory mechanisms operating in ocular tumors. First, microRNA-mediated regulation: Bellazzo et al. [73] demonstrated that miR-149-3p directly targets *DAB2IP* mRNA for degradation or translational repression, providing a precedent for post-transcriptional silencing despite elevated transcript levels. Second, differential protein stability: Dai et al. showed that *DAB2IP* undergoes ubiquitin-proteasome-mediated degradation via SCFFbw7 [84], potentially explaining low protein levels despite high mRNA. Third, compensatory transcriptional responses may occur when cells upregulate mRNA synthesis in response to protein loss through feedback loops. These findings underscore that transcriptome profiling alone provides incomplete information about functional protein expression, emphasizing the critical importance of orthogonal protein-level validation performed in our study.

Analysis of GSE62075 (uveal melanoma cell lines vs. normal melanocytes) showed no significant differential expression for any of the five genes. Importantly, the absence of significant differential expression in uveal melanoma cell lines compared with normal melanocytes highlights a key limitation of in vitro and in silico transcriptomic models, which often fail to reproduce the microenvironmental, metabolic, and differentiation-dependent regulation present in primary ocular tumors; this discrepancy further supports the involvement of post-transcriptional regulatory mechanisms underlying our protein-level findings. This null finding reflects well-documented limitations of immortalized cell line models, which undergo selective pressures during establishment and passage that may erase expression patterns present in primary tumors. Previous studies have documented extensive transcriptomic divergence between uveal melanoma cell lines and primary tumors [85,86], emphasizing that cell culture systems inadequately recapitulate the tumor microenvironment, stromal interactions, and cellular heterogeneity of native ocular malignancies. This negative result in cell lines paradoxically strengthens our primary findings by highlighting the necessity of studying fresh primary tumor specimens, as we have done.

Analysis of TCGA uveal melanoma data (*n* = 78 patients) validated clinical relevance beyond our institutional cohort. *GIPC1* emerged as an independent adverse prognostic factor in multivariate analysis, while *DAB2IP* showed protective associations. The reproducibility of GIPC1′s prognostic value across independent patient populations supports its potential utility as a biomarker, though prospective validation is required before clinical implementation.

### 4.9. Study Limitations and Future Directions

Several limitations warrant acknowledgment. First, our study is descriptive and correlative in nature, lacking functional validation experiments. We did not perform in vitro or in vivo manipulations (overexpression, knockdown, or knockout) of LRP2, CUBN, CAV1, GIPC1, or DAB2IP to establish causality between expression changes and tumor phenotypes. While our immunofluorescence data document expression patterns and public datasets provide prognostic associations, these observations alone cannot distinguish whether protein alterations are drivers of tumorigenesis or passenger events accompanying malignant transformation. Functional studies using patient-derived organoids, xenograft models, or CRISPR-mediated gene editing would be required to establish causal relationships and elucidate the precise mechanistic contributions of each protein to ocular tumor initiation, progression, and metastasis. Second, our institutional cohort comprises modest sample sizes per tumor subtype (*n* = 10 RB; *n* = 10 per melanoma subtype), limiting statistical power and generalizability. The single-institution design restricts assessment of inter-institutional variability and precludes evaluation across diverse ethnic and geographic populations. Third, the cross-sectional design captures expression at a single time point, precluding assessment of temporal dynamics during tumor evolution or treatment response. Longitudinal studies with serial biopsies would better establish whether expression changes are early events in tumorigenesis or late manifestations of progression.Fourth, while we integrated public transcriptomic datasets, large-scale proteomic repositories for ocular tumors remain unavailable, limiting opportunities for independent protein-level validation. The protein-mRNA discordances we observed underscore the importance of orthogonal validation, yet most public datasets provide only transcript-level information. Fifth, our quantitative immunofluorescence approach, while rigorously standardized, reflects relative rather than absolute protein abundance and does not capture post-translational modifications, subcellular compartmentalization, or protein–protein interactions that may modulate functional activity. Another limitation is the incomplete availability of clinical covariates (including age, sex, and anatomical site), which prevented multivariable adjustment in survival analyses. Future multicenter datasets with standardized metadata will be required to account for such confounders. In addition, future studies would benefit from spatially resolved profiling approaches, including multiplex immunofluorescence, imaging mass cytometry, and single-cell or spatial transcriptomic platforms, to localize protein expression to specific tumor, stromal, and vascular compartments and better characterize microenvironment-dependent regulation.

While we leveraged available public transcriptomic datasets, large-scale proteomic repositories for ocular tumors remain scarce, limiting opportunities for protein-level external validation. The observed protein-mRNA discordances highlight the urgent need for matched proteomic-transcriptomic profiling in the same specimens to systematically characterize post-transcriptional regulatory mechanisms. Future multicenter studies should employ integrated multi-omics approaches (proteomics, transcriptomics, epigenomics) with larger cohorts to confirm these findings. Additionally, functional validation in patient-derived organoid or xenograft models could clarify whether observed expression changes are drivers or passengers of tumorigenesis.

Taken together, these analyses position *GIPC1* as an independent prognostic biomarker and potential oncogenic driver in uveal melanoma, while *DAB2IP* functions as a protective tumor suppressor. *CAV1* appears to be context-dependent, being significant only in univariate settings. The discordance between transcriptomic and protein-level findings emphasizes the importance of integrative analyses and tumor-specific context when evaluating candidate biomarkers in ocular oncology.

## 5. Conclusions

This study delineates subtype-linked expression of five endocytic and signaling proteins across normal ocular tissue, RB, and uveal melanoma. LRP2 was uniformly reduced. CUBN was reduced in RB and epithelioid melanoma and retained or increased in other subtypes. CAV1 increased in epithelioid melanoma and declined in other subtypes. GIPC1 and DAB2IP were preserved in epithelioid melanoma and reduced in RB and mixoid or spindle melanomas. Integrated analyses associated higher GIPC1 and context-dependent CAV1 with poorer survival. DAB2IP appeared protective in multivariable models. Protein–transcript discrepancies underscore the value of multi-level profiling.

These findings nominate LRP2, CUBN, CAV1, GIPC1, and DAB2IP as complementary markers of differentiation state and tumor behavior in ocular oncology. Future studies should validate these signatures in larger multicenter cohorts. Spatial proteo-transcriptomics would refine cellular context. Functional assays should test Megalin–Cubilin nutrient handling, CAV1-mediated signaling, and GIPC1/DAB2IP pathway control. These efforts may improve risk stratification and support biomarker-guided therapeutic strategies for RB and uveal melanoma.

## Figures and Tables

**Table 1:** Antibodies used for immunofluorescence.

Antibodies		Catalog Number	Host	Dilution	Source
Primary	Anti-Lrp2/Megalin antibody	ab76969	Rabbit	1:250	Abcam, Cambridge, UK
	Human/Mouse/Rat Cubilin Antibody	#AF3700	Sheep	1:50	R&D Systems, Inc., Minneapolis, MN, USA
	Caveolin-1 (D46G3) XP^®^ Rabbit mAb	#3267S	Rabbit	1:300	Cell Signaling Technology (CST), Danvers, MA, USA
	Gipc1 Polyclonal antibody	14822-1-AP	Rabbit	1:100	Proteintech Group, Inc., Rosemont, IL, USA
	Dab2IP Polyclonal antibody	23582-1-AP	Rabbit	1:50	Proteintech Group, Inc., Rosemont, IL, USA
Secondary	Alexa Fluor^®^ 488 AffiniPure™ Donkey Anti-Rabbit IgG (H + L)	711-545-152	Donkey	1:300	Jackson ImmunoResearch Laboratories, Inc., West Grove, PA, USA
	Alexa Fluor^®^ 488 Affini-Pure™ Donkey Anti-Sheep IgG (H + L)	713-545-003	Donkey	1:300	Jackson ImmunoResearch Laboratories, Inc., West Grove, PA, USA

## Data Availability

The data supporting the findings of this study are available from the corresponding author upon reasonable request. Gene expression and survival data for *LRP2*, *CUBN*, *DAB2IP*, *GIPC1*, and *CAV1* in uveal melanoma (UVM) were obtained from publicly accessible databases, including the UCSC Xena platform (https://xenabrowser.net), The Cancer Genome Atlas (TCGA; https://www.genome.gov/Funded-Programs-Projects/Cancer-Genome-Atlas, accessed on 16 August 2025), the GTEx Portal (https://gtexportal.org/home/, accessed on 16 August 2025), and the Gene Expression Profiling Interactive Analysis tools GEPIA2 (http://gepia2.cancer-pku.cn/) and GEPIA3 (https://gepia3.bioinfoliu.com/). All resources were accessed on 30 July 2025. These databases provide transcriptomic and clinical data that support the differential expression and survival analyses presented in this study.

## Supplementary Materials

The following supporting information can be downloaded at: https://www.mdpi.com/article/10.3390/cancers17233785/s1, Figure S1: Negative control immunofluorescence staining (secondary antibody only); Figure S2: Positive control validation in mouse kidney tissue; Figure S3: Positive control validation in mouse kidney tissue; Figure S4: ImageJ quantification pipeline for immunofluorescence analysis.

## Abbreviations

The following abbreviations are used in this manuscript:

AKT	AKT serine/threonine kinase
ANOVA	Analysis of variance
ASK1	Apoptosis signal-regulating kinase 1
CAV1	Caveolin-1
CI	Confidence interval
CTRL	Control
CUBN	Cubilin
DAB2IP	Disabled homolog 2–interacting protein
DAPI	4′,6-diamidino-2-phenylindole
EMT	Epithelial–mesenchymal transition
ERK	Extracellular signal-regulated kinase
FFPE	Formalin-fixed, paraffin-embedded
GDC	Genomic Data Commons
GEO	Gene Expression Omnibus
GEPIA2	Gene Expression Profiling Interactive Analysis 2
GEPIA3	Gene Expression Profiling Interactive Analysis 3
GIPC1	GIPC PDZ domain-containing protein 1
H&E	Hematoxylin and eosin
HPF	High-power field
HR	Hazard ratio
IF	Immunofluorescence
IHC	Immunohistochemistry
JNK	c-Jun N-terminal kinase
Log-rank	Mantel–Cox test
LRP2	Low-density lipoprotein receptor-related protein 2 (megalin)
NA	Numerical aperture
NF-κB	Nuclear factor kappa-B
NRP1	Neuropilin-1
OS	Overall survival
PBS	Phosphate-buffered saline
PDGFR	Platelet-derived growth factor receptor
PI3K	Phosphoinositide 3-kinase
RB	Retinoblastoma
RhoA	Ras homolog family member A
SD	Standard deviation
TCGA	The Cancer Genome Atlas
UM	Uveal melanoma
UVM	Uveal Melanoma (TCGA cohort code)
