# Supplementary material for: Linking Megalin, Cubilin, Caveolin-1, GIPC1 and Dab2IP Expression to Ocular Tumorigenesis: Profiles in Retinoblastoma, Choroidal Melanoma, and the Normal Human Eye
Source: Cancers (Basel). 2025 Nov 26;17(23):3785. doi: 10.3390/cancers17233785 (PMC12691367; doi:10.3390/cancers17233785)
Supplement: Supplementary file 1 [file cancers-17-03785-s001.zip › cancers-3977797-supplementary.pdf]

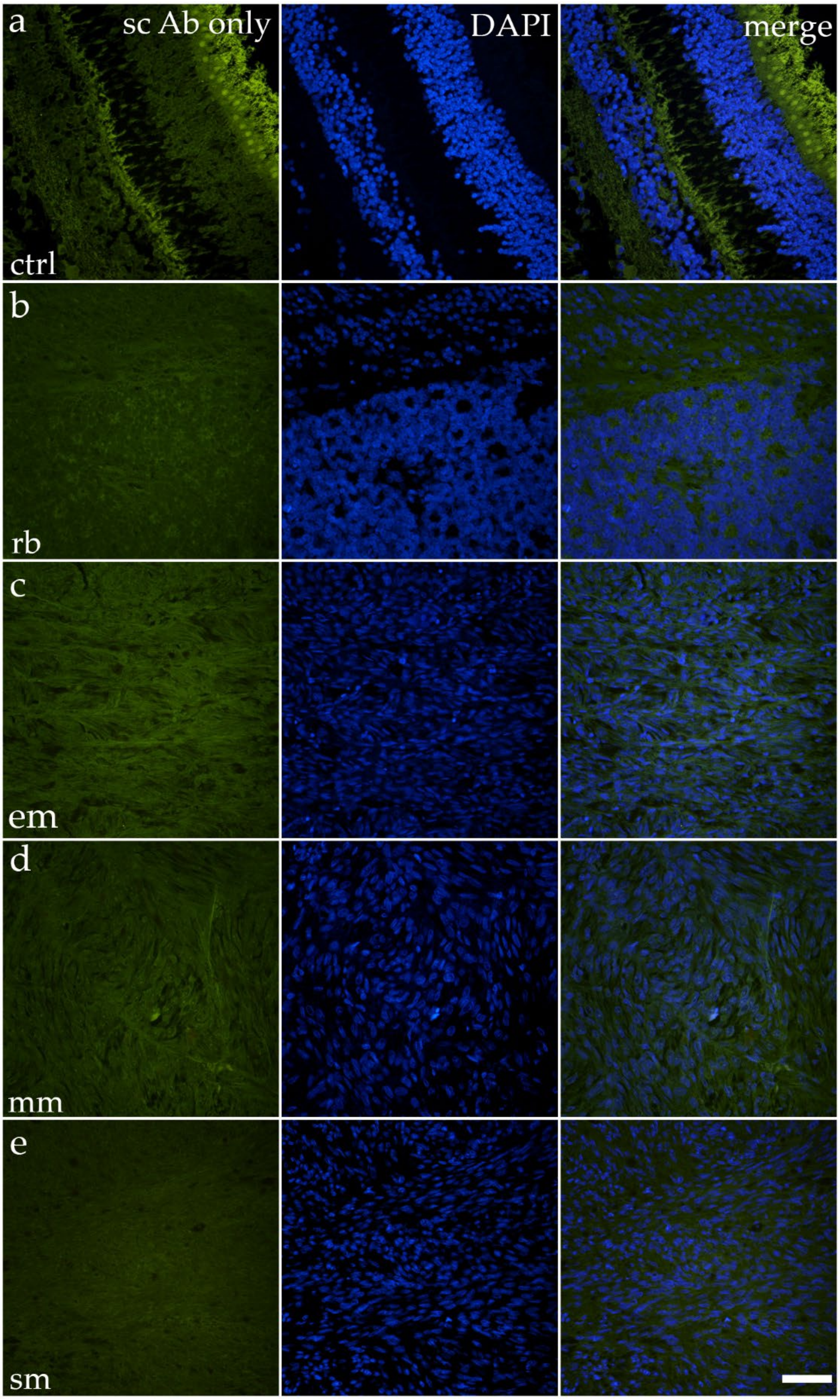

Figure S1. Negative control immunofluorescence staining (secondary antibody only). Representative images of negative control sections processed with secondary antibody only (omission of primary antibody) across all tissue types. Left column: Green fluorescence channel (Alexa Fluor 488) showing absence of specific signal. Middle column: DAPI nuclear counterstain. Right column: Merged channels.(a) Control retinal tissue (ctrl), (b) retinoblastoma (rb), (c) epithelioid melanoma (em), (d) myxoid melanoma (mm), and (e) spindle melanoma (sm). Note the absence of specific green fluorescence signal in all tissue types when primary antibody is omitted, demonstrating

antibody specificity and minimal non-specific binding of the secondary antibody. Only faint autofluorescence is observed, particularly in the control retinal tissue (a), which is characteristic of lipofuscin-containing retinal pigment epithelium and photoreceptor outer segments. The low background signal (<5% of positive control intensity) confirms the specificity of the primary antibodies used in this study. Scale bar: 50 μm. Magnification: ×40.

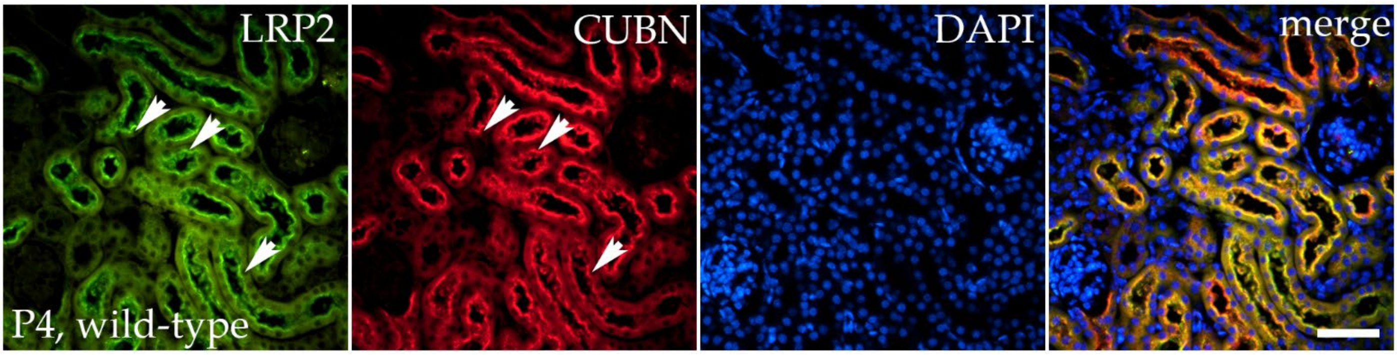

Figure S2. Positive control validation in mouse kidney tissue. Immunofluorescence staining of postnatal day 4 (P4) wild-type mouse kidney demonstrating expected expression patterns for LRP2 and CUBN. From left to right: LRP2 (green, Alexa Fluor 488), CUBN (red, Alexa Fluor 594), DAPI nuclear stain (blue), and merged channels. Both LRP2 (arrowheads) and CUBN (arrowheads) show strong, specific apical membrane staining in proximal tubule epithelial cells, consistent with their established co-localization and function as multiligand endocytic receptors in renal tubular reabsorption. The characteristic brush border pattern (intense apical staining with minimal cytoplasmic or basal signal) confirms antibody specificity and proper subcellular localization. In the merged image, co-expression of LRP2 and CUBN produces yellow-orange fluorescence at the apical membrane, reflecting their known functional partnership in receptor-mediated endocytosis. Scale bar: 50 μm. Magnification: ×40.

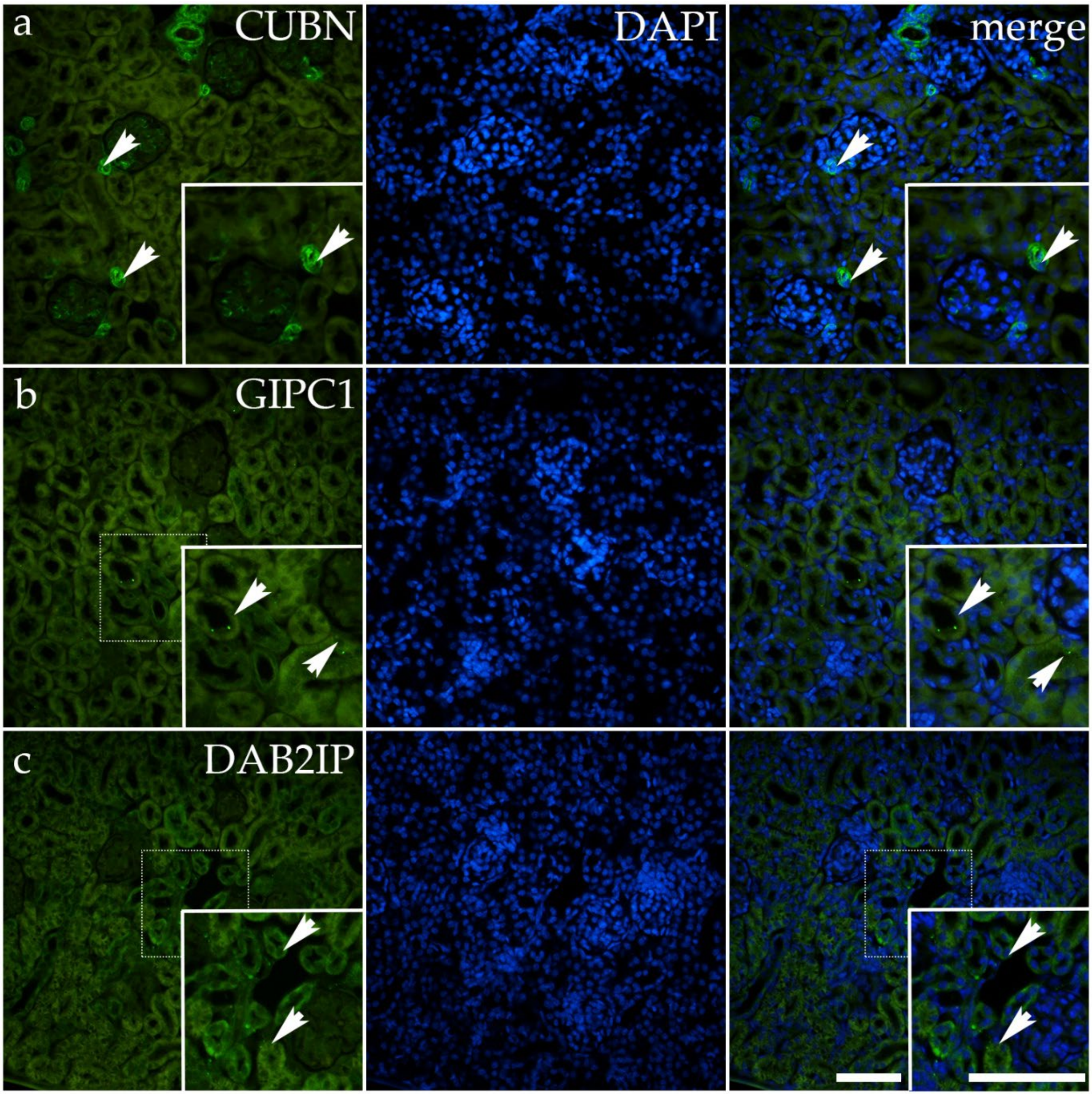

Figure S3. Positive control validation in mouse kidney tissue. Immunofluorescence staining of postnatal day 4 (P4) wild-type mouse kidney demonstrating expected expression patterns for (a) CUBN, (b) GIPC1, and (c) DAB2IP. Left column: Green fluorescence (Alexa Fluor 488); middle column: DAPI nuclear counterstain; right column: merged channels. Insets show higher magnification of boxed regions.(a) CUBN shows apical membrane staining (arrowheads) in proximal tubule epithelial cells, consistent with its established role as an endocytic receptor. Signal intensity is moderate with characteristic brush border localization. (b) GIPC1 demonstrates diffuse cytoplasmic expression in renal tubular epithelial cells (arrowheads in inset). The staining pattern is consistent with GIPC1's role as an intracellular adaptor protein involved in receptor trafficking and vesicular transport. (c) DAB2IP shows cytoplasmic expression in tubular epithelial cells (arrowheads in inset), with a granular/punctate distribution pattern consistent with its function as a cytoplasmic scaffold protein.While these kidney sections show lower signal intensity compared to ocular tissues, the specific cellular localization patterns confirm antibody specificity. The cytoplasmic distribution of GIPC1 and DAB2IP contrasts with the apical membrane localization of CUBN, reflecting their distinct subcellular functions. Scale bars: 50 μm (main panels and insets as indicated).

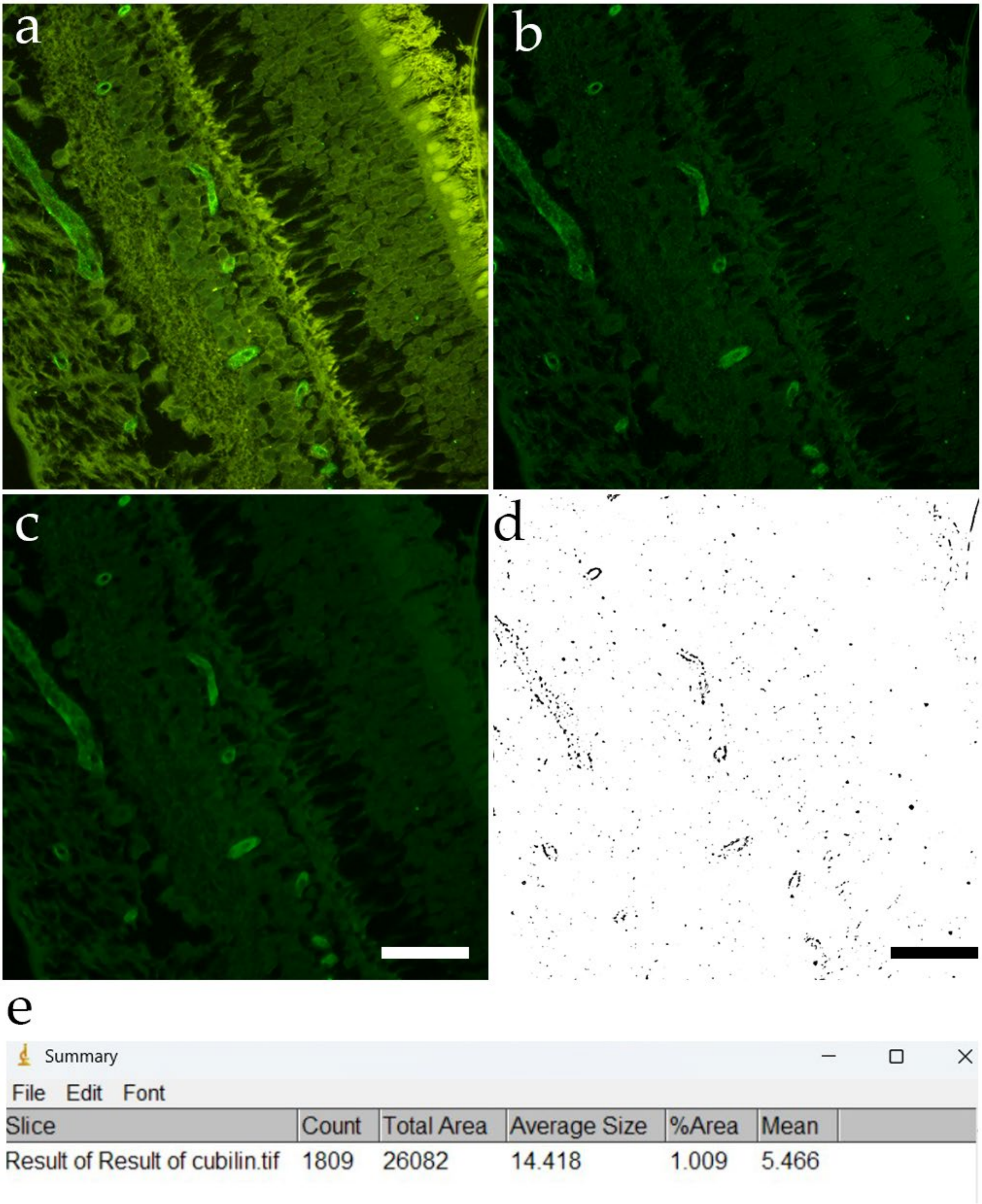

Slice	Count	Total Area	Average Size	%Area	Mean
Result of Result of cubilin.tif	1809	26082	14.418	1.009	5.466

Figure S4. ImageJ quantification pipeline for immunofluorescence analysis. Representative image processing workflow for CUBN immunofluorescence quantification in control retinal tissue, demonstrating the standardized ImageJ analysis protocol. (a) Original raw immunofluorescence image showing CUBN expression (green, Alexa Fluor 488). Strong apical membrane staining is visible in the retinal epithelium. (b) Image after red counter-signal subtraction to eliminate fluorescence spillage from other channels, isolating the specific green fluorescence. (c) Background-subtracted image following application of median filter (7.0-pixel radius). This step removes noise while preserving genuine positive signal, isolating specific immunoreactivity from background fluorescence. (d) Binary image after threshold application using the triangle algorithm and conversion to 8-bit format. Black particles represent positive fluorescent signal above threshold; white background

represents negative areas. The "Analyse Particles" function in ImageJ quantifies these regions.(e) ImageJ summary output showing quantification results: Count: 1809 (number of discrete positive particles detected), Total Area: 26082 pixels (sum of all positive signal area); Average Size: 14.418 pixels per particle; %Area: 1.009 (percentage of total image area occupied by positive signal); Mean: 5.466 (mean intensity value. The %Area value (1.009%) represents the proportion of the image occupied by CUBN-positive fluorescent signal and is the primary metric used for statistical comparisons across tissue groups. This standardized, semi-automated approach ensures objective, reproducible quantification across all samples. Scale bars: 50 μm.
